# A supported self-help for recurrent depression in primary care; An economic evaluation alongside a multi-center randomised controlled trial

**DOI:** 10.1371/journal.pone.0208570

**Published:** 2018-12-19

**Authors:** Karolien E. M. Biesheuvel-Leliefeld, Judith E. Bosmans, Sandra M. A. Dijkstra-Kersten, Filip Smit, Claudi L. H. Bockting, Digna J. F. van Schaik, Harm W. J. van Marwijk, Henriette E. van der Horst

**Affiliations:** 1 Department of General Practice and Elderly Care Medicine and EMGO^+^ Institute for Health and Care Research, VU University Medical Center, Amsterdam, The Netherlands; 2 Department of Health Sciences and the EMGO Institute for Health and Care Research, Faculty of Earth and Life Sciences, VU University, Amsterdam, The Netherlands; 3 Netherlands Institute of Mental Health and Addiction, Utrecht, The Netherlands; 4 Department of Clinical Psychology and EMGO^+^ Institute for Health and Care Research, VU University and VU University Medical Center, Amsterdam, The Netherlands; 5 Department of Epidemiology and Biostatistics, EMGO^+^ Institute for Health and Care Research, VU University Medical Center, Amsterdam, The Netherlands; 6 Department of Clinical Psychology and Experimental Psychopathology, University of Utrecht, Utrecht, The Netherlands; 7 Department of Psychiatry and the EMGO+ Institute for Health and Care Research, VU University Medical Center, Amsterdam, The Netherlands; 8 Manchester Academic Health Sciences Centre and NIHR School for Primary Care Research, Manchester, United Kingdom; Public Library of Science, UNITED KINGDOM

## Abstract

**Background:**

Major depression is a prevalent mental disorder with a high risk of relapse or recurrence. Only few studies have focused on the cost-effectiveness of interventions aimed at the prevention of relapse or recurrence of depression in primary care.

**Aim:**

To evaluate the cost-effectiveness of a supported Self-help Preventive Cognitive Therapy (S-PCT) added to treatment-as-usual (TAU) compared with TAU alone for patients with a history of depression, currently in remission.

**Methods:**

An economic evaluation alongside a multi-center randomised controlled trial was performed (n = 248) over a 12-month follow-up. Outcomes included relapse or recurrence of depression and quality-adjusted-life-years (QALYs) based on the EuroQol-5D. Analyses were performed from both a societal and healthcare perspective. Missing data were imputed using multiple imputations. Uncertainty was estimated using bootstrapping and presented using the cost-effectiveness plane and the Cost-Effectiveness Acceptability Curve (CEAC). Cost estimates were adjusted for baseline costs.

**Results:**

S-PCT statistically significantly decreased relapse or recurrence by 15% (95%CI 3;28) compared to TAU. Mean total societal costs were €2,114 higher (95%CI -112;4261). From a societal perspective, the ICER for relapse or recurrence was 13,515. At a Willingness To Pay (WTP) of 22,000 €/recurrence prevented, the probability that S-PCT is cost-effective, in comparison with TAU, is 80%. The ICER for QALYs was 63,051. The CEA curve indicated that at a WTP of 30,000 €/QALY gained, the probability that S-PCT is cost-effective compared to TAU is 21%.

**Conclusions:**

Though ultimately depending on the WTP of decision makers, we expect that for both relapse or recurrence and QALYs, S-PCT cannot be considered cost-effective compared to TAU.

## Introduction

Major depressive disorder (MDD) is a prevalent mental disorder that often runs an intermittent lifelong course[1], is associated with a high risk of relapse or recurrence[2] and with frequently incomplete remission between episodes[3–5]. It is considered to be among the most disabling illnesses[6], and negatively affects many aspects of life[7–9].

Largely due to its recurrent nature, the economic consequences of MDD are substantial [10–13]. It has been estimated that 1–2% of national healthcare expenses in Western countries is spent on the treatment of depressive disorders[14,15]. Important factors contributing to the considerable healthcare costs associated with depression are the high prevalence, early age of onset and large risk of relapse or recurrence. However, the majority of costs associated with MDD are due to loss of productivity [14,16–18]. Therefore, effective preventive interventions may be beneficial from the viewpoint of patients and society [19,20]. However, these interventions are mostly offered in secondary care, often relying on the intensive use of therapist’s time, and, therefore, they are costly. A minimally supported self-help intervention may help to overcome this problem.

A randomised clinical trial by Biesheuvel et al.[21] showed superior effect of a supported Self-help Preventive Cognitive Therapy (S-PCT) plus treatment-as-usual (TAU) over TAU alone in preventing relapse or recurrence in patients with a history of depressive episodes. Due to the current economic down-turn and the scarcity of resources available for healthcare, information on the cost-effectiveness of interventions is highly relevant for decision-makers. Alongside this trial, we now investigate the cost-effectiveness of S-PCT compared with TAU from both a societal and a healthcare perspective, in patients with a history of depression.

## Methods

### Design

An economic evaluation was performed alongside a pragmatic randomised controlled trial with two parallel groups of participants. The design of this study is described in more detail elsewhere [22]. The study is registered in the Dutch Trial Register, www.trialregister.nl, NTR3001.

### Ethics

The Medical Ethics Committee of the Vrije Universiteit medical center (VUmc) approved the study protocol (S1 Protocol) and all participants provided written informed consent.

### Terminology

To describe the course of depression, we use the operational criteria of Frank et al.[23]. According to these criteria, the course of depression is described as a series of disease stages in which a patient can move from a symptom-free stage, to a stage characterised by some symptoms but not meeting the diagnostic criteria, to a stage with the full-blown disorder, after which the patient can go into remission. When a patient stays in remission for a minimum of six months, he or she is considered to be recovered. A relapse is defined as a depressive episode that occurs during remission and before recovery, while a recurrence is defined as a depressive episode that occurs after recovery.

### Treatment allocation

Once participants had provided informed consent, they received the Structured Clinical Interview for DSM-IV Axis 1 disorders 3.0 [24] (SCID-1 3.0) to assess eligibility criteria. When participants were eligible, randomisation was conducted by an independent statistician using computer-generated random numbers in blocks of size two. Participants were randomised on the order in which their baseline SCID-1 3.0- was conducted by the researchers. Randomisation was stratified by the number of previous depressive episodes (2–3 episodes versus ≥4 episodes) because the number of previous episodes is associated with relapse or recurrence [25]. Randomisation was concealed from the assessors who conducted interviews during the observation period, as they were not informed about the participants’ randomisation status, and participants were requested not to disclose randomisation status to the assessors.

### Blinding

Interviewers were blind for randomization status of the participants during all measurements. Due to the nature of the intervention, it was not possible to blind the participants. At the start of each interview, participants were asked not to reveal their allocation status to the interviewers.

### Participants

Participants were recruited through general practices and mental health care services in the Netherlands. To be included in the trial, participants had to a) be 18 years or older, b) be in remission of recurrent MDD for at least two months, but no longer recovered than five years according to the SCID-1 3.0) and c) have experienced two or more previous episodes of MDD. The SCID-I 3.0 interview was conducted by telephone by trained researchers and psychologists. Exclusion criteria were severe cognitive impairments, current or past mania, hypomania or psychosis, current alcohol or drug abuse, or insufficient mastery of the Dutch language.

### Counsellors

Twenty-four counsellors (primary care mental health nurses and psychologists) were trained to support the intervention. The psychologists were non-specialised psychologists (no postdoctoral training in clinical interventions). All counsellors attended a one-day training delivered by experienced clinical psychologists, who developed the intervention and, therefore, had an intimate knowledge of PCT. To detect competence issues, audiotaped telephone contacts with two participants of each counsellor were evaluated during a one-day supervision with the trainer(s) before the actual start of the trial. During the trial, counsellors could contact the trainers at any time for additional questions and feedback.

### Intervention

The self-help preventive cognitive therapy is a manualised PCT-based bibliotherapy consisting of a printed self-help book with eight modules and minimal guidance [26]. It is based on an effective face-to-face PCT [27,28] and mobile PCT[29]. PCT for the prevention of depression is an adapted type of Cognitive Therapy (CT) for acute depression[30] and aims to prevent relapse or recurrence in remitted patients with a history of depressive episodes. Like regular CT, PCT follows a fixed structure, with agenda setting, review of homework, explanation of the rationale of each session, and the assignment of homework. Participants complete one module per week. Each module includes both reading plus assignments to be completed in approximately 60 minutes. During the first meeting (by phone or face-to-face), the counsellor explained the rationale of S-PCT and the planning for the coming week. Each week the counsellor contacted the participant by phone to evaluate progress and understanding. This call was strictly protocolled and was designed to last no longer than 15 minutes. The nature of the contact was solely to support the participant, and not to actively engage in a therapeutic relationship. Adherence to the intervention protocol was assessed using a checklist. Each week, the counsellor completed this checklist with 4 items; (1) the number of that week’s module (1–8), (2) did the participants read the literature of that week (yes/no plus reason), (3) did the participant do the assignments (yes/no plus reason) and (4) time spent on the call (minutes).

### Treatment-as-usual (TAU)

There were no restrictions to type of TAU. Care providers were not aware of randomization status unless participants informed them. Current TAU guidelines suggest continuation of antidepressant medication (ADM) for at least 6 months after remission[31]. People at significant risk of relapse or recurrence, should be offered individual Cognitive (Behavourial)Therapy (C(B)T) or Mindfulness-Based Cognitive Therapy (MBCT). TAU (e.g. ADM or psychiatric counselling) was recorded using the Trimbos and iMTA questionnaire for costs associated with Psychiatric Illnesses (TiC-P)[32].

### Sample size

We combined findings from previous research [33,34] and assumed a mean relapse or recurrence rate of 40% during the 12-months observation period versus 60% in the controls. To detect this 20% risk-reduction in a 2-sided test at α = 0.05 and a power of 1-β = 0.80, 107 participants in each condition were required. Compensating for loss to follow-up of 10% over the whole 12-month observation period, required at least (107/0.90 =) 119 participants at baseline in each trial arm. Our own experience with randomization of patients at general practice level [35,36] indicates that clustering of patients within practices has no impact on the power of the trial. Therefore, we did not take clustering effects into account.

### Clinical outcome measures

#### Relapse or recurrence

Primary outcome was the incidence of relapse or recurrence of depression over the 12 months follow-up period. To reduce recall bias, the telephone SCID-1 3.0 was conducted at both 6 and 12 months and combined into a single outcome (0 = no relapse or recurrence, 1 = relapse or recurrence).

#### Quality of life

*Health related quality of life* (HRQoL) was evaluated using the Dutch translations of the EuroQol-5D (three levels) questionnaire (EQ-5D-3L)[37]. The EQ-5D measures Health Related Quality of Life (HRQoL) on five dimensions (mobility, self-care, usual activities, pain/discomfort and anxiety and depression), combined into one outcome. Each dimension is rated at three levels corresponding to whether a respondent has no problems, moderate or extreme problems. Utility scores for the EQ-5D health states were estimated using preference weights obtained from the Dutch population [38]. Quality-adjusted life-years (QALYs) were calculated by multiplying the utility scores belonging to a health state by the amount of time spent in this health state using linear interpolation between time points.

### Costs

Costs were assessed every 3 months during the 12-month follow-up period and categorised into 8 groups: primary care costs (general practice visits), secondary care costs (including consultations, day care and hospital days), mental health care costs (sessions with psychologists and/ or psychiatrists in primary and secondary care), home care costs (formal help with domestic tasks), medication costs, costs due to lost productivity, informal care costs (help from family and friends) and intervention costs (costs of S-PCT).

#### Health care utilization costs

The TiC-P [32] was used to assess the utilization of formal health care services (primary care, secondary medical care,) and informal care during the last 3 months. Costs were computed by multiplying the units of health care use (visits, consultations, sessions, hospital days) by the standard cost prices of these services as reported in the Dutch costing guideline[39]. Costs of medication were valued using daily defined doses (DDD) and the cost prices of the Royal Dutch Society for Pharmacy [40] to which the pharmacist’s dispensing costs were added. All costs were indexed for the year 2013.

#### Lost productivity costs

The TiC-P was used to measure productivity losses due to both absenteeism (absent from work) and presenteeism (less productive at work). Costs of absenteeism from paid work were calculated according to both the human capital-, and the friction cost approach, using the mean age-, and sex specific income of the Dutch population[41]. In the human capital method, any hour not worked counts as an hour lost. By contrast, according to the friction cost approach, a sick employee is replaced after a certain amount of time (the friction period; currently 160 days) after which there are no longer productivity losses, because the absent employee will be replaced by someone else. Costs of presenteeism were calculated by asking participants how many working hours should have been replaced due to less productivity at work. Lost productivity due to presenteeism was valued using the mean age-, and sex specific income of the Dutch population[41]. Costs of productivity losses due to absenteeism from unpaid work and informal care were calculated using the standard wage of a professional housekeeper[42]. Discounting was unnecessary, because neither costs nor benefits were recorded beyond 12 months.

#### Cost price supported self-help PCT

A cost price for S-PCT was calculated using a bottom-up approach. Cost price for S-PCT totaled €388.15 per recipient. The average costs of screening the participants was estimated to be €46.12, consisting of 45 minutes of time by the counsellor (€35.92) and the time spent by the participant to complete the interview (€10.20). Costs that were included in the cost price calculation of S-PCT were the printing costs of the bibliotherapeutic booklet including sending it by mail (€2.13), the costs of support and administration time invested by the counsellor (€ 143.67), telephone costs by the counsellor (€5.40) the time costs by participants (on the phone €40.80 + assignments €54.40) and the per-participants costs associated with training (€63.75) and supervising (€31.88) the counsellor. We used the time spent and the Dutch tariff (hour rate of psychologist) to calculate support costs and administration time of the counsellor. We deliberately included time costs by participants, because in a self-help intervention, the idea is that much of the therapeutic work is done by the participants themselves.

### Missing data

Missing data on costs and outcomes were imputed using multiple imputations with chained equations (MICE) using predictive mean matching and fully conditional specification in STATA 12 [43,44]. An imputation model was created that contained variables related to missing data and variables that significantly differed at baseline between the groups. Ten imputed datasets were created, resulting in loss of efficiency of less than 2.5% for all outcomes[43]. The 10 imputed datasets were analyzed separately and the results of the analyses were pooled using Rubin’s rules[45].

### Statistical analysis

The statistical analyses were conducted according to the intention-to-treat (ITT) principle. In the cost-effectiveness analysis (CEA), we estimated Incremental Cost-Effectiveness Ratios (ICERs) as the ratio of the difference in mean costs divided by the difference in mean effects on depressive relapse or recurrence between S-PCT and TAU. In the cost-utility analysis (CUA), the ICER was defined by the ratio of the difference in costs between the S-PCT and the TAU group by the difference in QALYs. Clinical effects were adjusted for depressive symptoms at baseline, which is a main predictor of relapse or recurrence. Therefore, we added baseline QIDS scores tot the Poisson regression model. Cost differences were adjusted for baseline costs. Therefore, we added baseline costs to the regression model.

Seemingly unrelated regression was used to estimate differences in costs and effects while accounting for potential correlation between costs and effects [46]. Costs generally have a highly skewed distribution. Therefore, non-parametric bootstrapping with 5000 replications was used to estimate bias-corrected and accelerated confidence intervals around cost differences[47] and to estimate uncertainty surrounding the ICERs. The bootstrapped cost-effect pairs were plotted on a cost-effectiveness plane [48] and used to estimate cost-effectiveness acceptability curves (CEACs). CEACs show the probability that the intervention is cost-effective in comparison with the control treatment for a range of ceiling ratios. The ceiling ratio is defined as the amount of money society is willing to pay (WTP, λ) to gain one unit of effect [49].

In the CEA, WTP-thresholds that were associated with probabilities of 80% and 95%, that the intervention was cost-effective compared to TAU, were assessed. In the CUA, the probabilities that the intervention was cost-effective in comparison with TAU at the commonly used WTP thresholds of €20.000 and €30.000 were assessed [50].

All analyses were performed with STATA (version 12) and SPSS (IBM SPSS Statistics 20).

### Sensitivity analyses

Two sensitivity analyses were performed. The first sensitivity analysis was performed from a health care perspective (HC). From a healthcare perspective, which is used in countries like the United Kingdom by the National Institute for Health and Clinical Excellence[31] (NICE), only direct healthcare costs are included, thus excluding costs related to informal care and costs related to lost productivity. In the per-protocol analysis (PP), statistical analysis was restricted to patients who completed at least 80% if the intervention (n = 101 out of 124 intervention participants).

## Results

### Participants flow and recruitment

**Fig 1:**
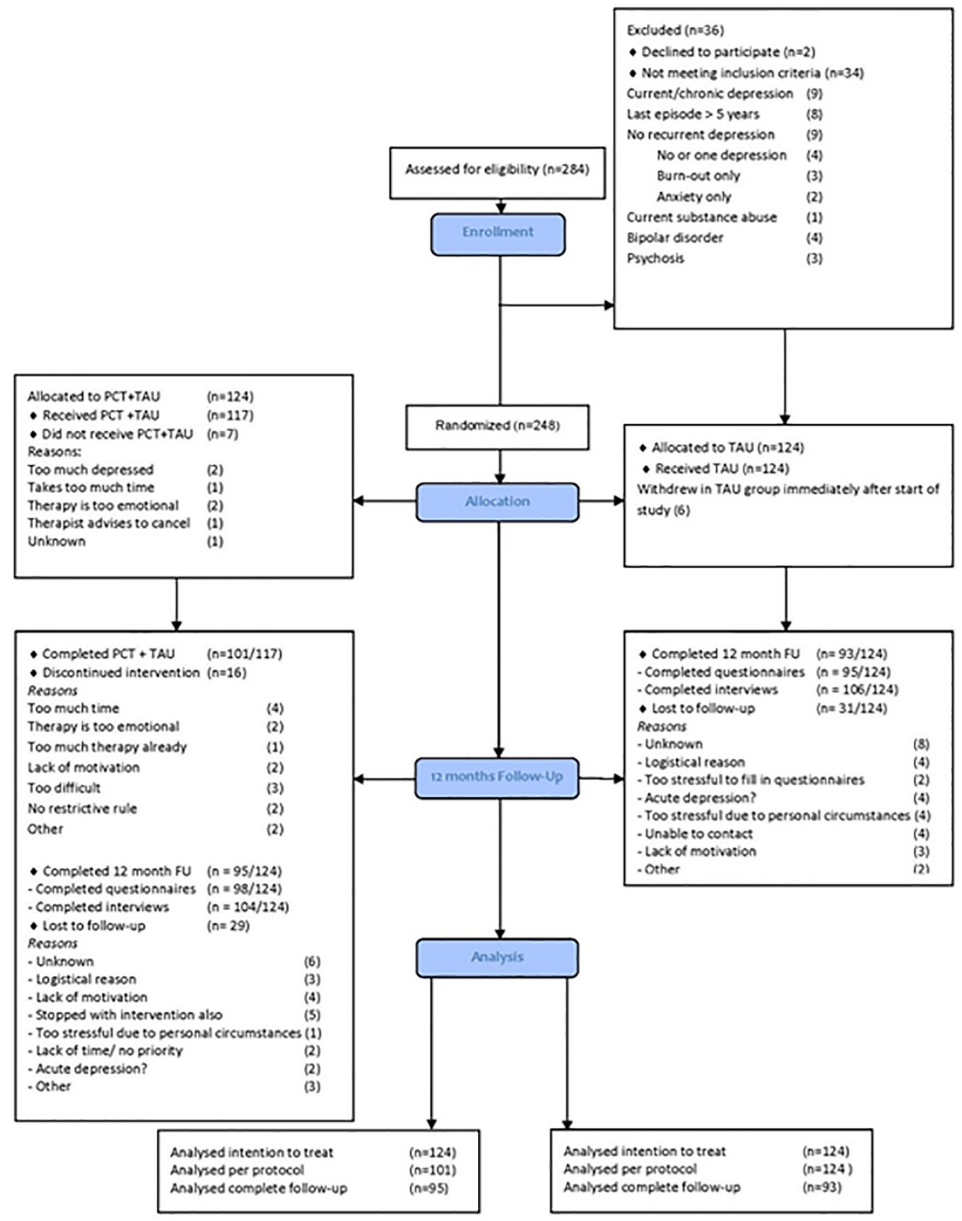
CONSORT flow diagram.

Details of enrolment are shown in Fig 1, according to the CONSORT recommendations (S1 Checklist). Recruitment took place between September 2012 and April 2014. Twenty-two databases of primary care practices and 4 databases of specialised mental health care practices were screened for eligible patients. This led to the selection of 5,489 potential participants who received a short information letter. Finally, 248 patients met all inclusion-criteria and signed informed consent. They were randomly allocated to the S-PCT group (124) or to the TAU group (124). Complete effect data over 12 months follow-up were available for 81.5% (101/124 participants). Complete cost data over 12 months follow-up were available for 79.8% (99/124) of the S-PCT patients and 75.8% (94/124) of the TAU patients. Participants without complete cost data experienced significantly more fatigue (0.60, 95%CI: 0.09;1.10) at baseline. More information about the pattern of missing data can be found in the article by Biesheuvel et al. [21].

### Baseline characteristics

In Table 1, baseline socio-demographic, clinical characteristics and costs of the ITT group are presented. Costs at baseline were assessed over the last three months.

**Table 1:** Baseline characteristics and societal costs by allocation group.

Characteristics	S-PCT (n = 124)	TAU (n = 124)	All (n = 248)
Age, mean (SD^1^; range)	48.6 (11.9; 20–76)	48.8 (11.4; 24–77)	48.7 (11.7; 20–77)
Females, n (%)	89 (71.8%)	84 (67.7%)	173 (69.8%)
Number of previous episodes, n (%)			
- 2 or 3	66 (53.2%)	62 (49.9%)	64 (51.6%)
- 4 or more	58 (46.8%)	62 (50.1%)	60 (48.4%)
Marital status, n (%) (with partner)	80 (64.9%)	80 (64.9%)	80 (64.9%)
Education^2^, n (%) (high education)	53 (42.7%)	44 (35.5%)	48 (39.1%)
Age of onset, mean (SD^1^)	28.2 (11.4)	27.5 (12.3)	27.8 (11.9)
Depressive symptoms (QIDSsr), mean (SD^1^)	9.6 (4.8)	8.9 (5.0)	9.3 (4.9)
Quality of life			
- Mental health (SF12, mean (SD^1^)	53.6 (12.2)	53.5 (11.6)	53.5 (11.9)
- Physical health (SF12, mean (SD^1^)	59.4 (11.4)	57.6 (11.7)	58.5 (11.6)
- EQ-5D (0–1), mean (SD^1^)	0.77 (0.21)	0.78 (0.20)	0.77 (0.20)
Comorbid psychopathology (4DSQ)			
- Anxiety (0–24), median (IQR)	2 (4)	1 (5) 00)	2 (4)
- Distress (0–32), mean (SD^1^)	13.0 (7.6)	12.7 (8.0)	12.8 (7.8)
- Somatization (0–32), mean (SD^1^)	8.1 (5.5)	8.9 (5.7)	8.5 (5.6)
Pain (MPQ), median (IQR)	0 (4)	0 (6)	0 (6)
Fatigue (FSS; 1–7), mean (SD^1^)	3.8 (1.5)	3.9 (1.6)	3.8 (1.6)
Self-efficacy (GSES; 10–40), mean (SD^1^)	28.6 (5.9)	28.3 (6.2)	28.4 (6.0)
ADM use past 3 months, %	51.8%	56.7%	54.2%
Societal costs^3^ (SD^1^)	1,620 (3,370)	1,185 (2,405)	1,406 (2,944)

TAU, treatment-as-usual; SD, standard deviation; ADM, anti-depressant medication; MPQ, MacGill Pain Questionnaire; FSS, Fatigue Severity Scale; GSES, General Self Efficacy Scale; EQ, EuroQol; QIDSsr, Quick Inventory of Depressive Symptoms self-report; 4DSQ, Four Dimensional Symptom Questionnaire; IQR, Interquartile range.

^1^ Standard deviations were computed from the standard errors: (sd = sqrt (_b[var^2^] -_b[var]*_b[var]))

^2^ Education is defined as bachelor’s or master’s degree

^3^ Mean societal costs per person over three months prior to baseline, euros (€)

### Clinical outcomes

Clinical effects are presented in Table 2. In the S-PCT group, 44 participants (35%) experienced a relapse or recurrence into a depression compared to 62 participants (50%) in the TAU group over 12 months. This difference was statistically significant (risk difference 0.15, 95%CI 0.03;0.28). The mean pooled difference in QALYs in the S-PCT group compared to the TAU group after 12 months was 0.03 (95%CI 0.0006;0.07).

**Table 2:** Multiply imputed pooled clinical outcomes and costs over 12-month follow-up^a^.

	S-PCT (n = 124)	TAU (n = 124)	Difference	95%CI^b^
**Clinical outcomes**				
Relapse or recurrence rate	0.35	0.50	0.15^c^	0.03;0.28
QALY	0.80	0.78	0.03^c^	0.0006;0.07
**Annual costs (2013, euros)**^c^				
- Primary care, mean (SD)	648 (89)	517 (72)	131	-107;378
- Secondary care, mean (SD)	1,680 (462)	871 (250)	810	-40;2246
- Mental health care, mean (SD)	581 (85)	626 (110)	-44	-351;179
- Home care, mean (SD)	127 (52)	117 (34)	10	-90;148
- Medication, mean (SD)	291 (34)	215 (26)	77	2.8;172
- Lost productivity, mean (SD)	3,919 (775)	2,538 (636)	1,381	-544;3148
*absenteeism*, mean (SD)	*2*,*648* (521)	*1*,*411* (468)	*1*,*236*	*-119;2376*
*presenteeism*, mean (SD)	*1*,*271* (293)	*1*,*127* (326)	*144*	*-772;1086*
Informal care, mean (SD)^d^	260 (36)	181 (25)	80	9;169
Intervention costs, mean (SD)	388 (39)	0 (0)	388	n/a
**Total costs, unadjusted**, mean (SD)	**7,897 (1.015)**	**5,065 (960)**	**2,832**	**479;5497**

S-PCT; supported self-help preventive cognitive therapy, QALY; Quality-Adjusted Life-Years, TAU; treatment-as-usual.

^a^ Presented are means and mean differences

^b^ 95% confidence intervals obtained by bias corrected and accelerated bootstrapping

^c^ Annual costs per person, measured at the end of each three-month period during 12 months follow-up

^d^ Informal care; care from family and friends

### Costs

Table 2 presents the multiply imputed and pooled cumulative annual costs in the S-PCT and TAU group over 12 months. After adjustment for baseline costs, the difference in total costs was €2,114 (95%CI -112;4261). For all cost-categories, mean costs for in the S-PCT group were higher than in the TAU group, except for costs of mental health care. There were no patients in the S-PCT group, nor in the TAU group who were absent for more than 160 subsequent days during the 12-month follow-up. Therefore, results of the human capital approach equaled the friction cost approach and are not reported separately.

### Cost-effectiveness; primary results

**Fig 2:**
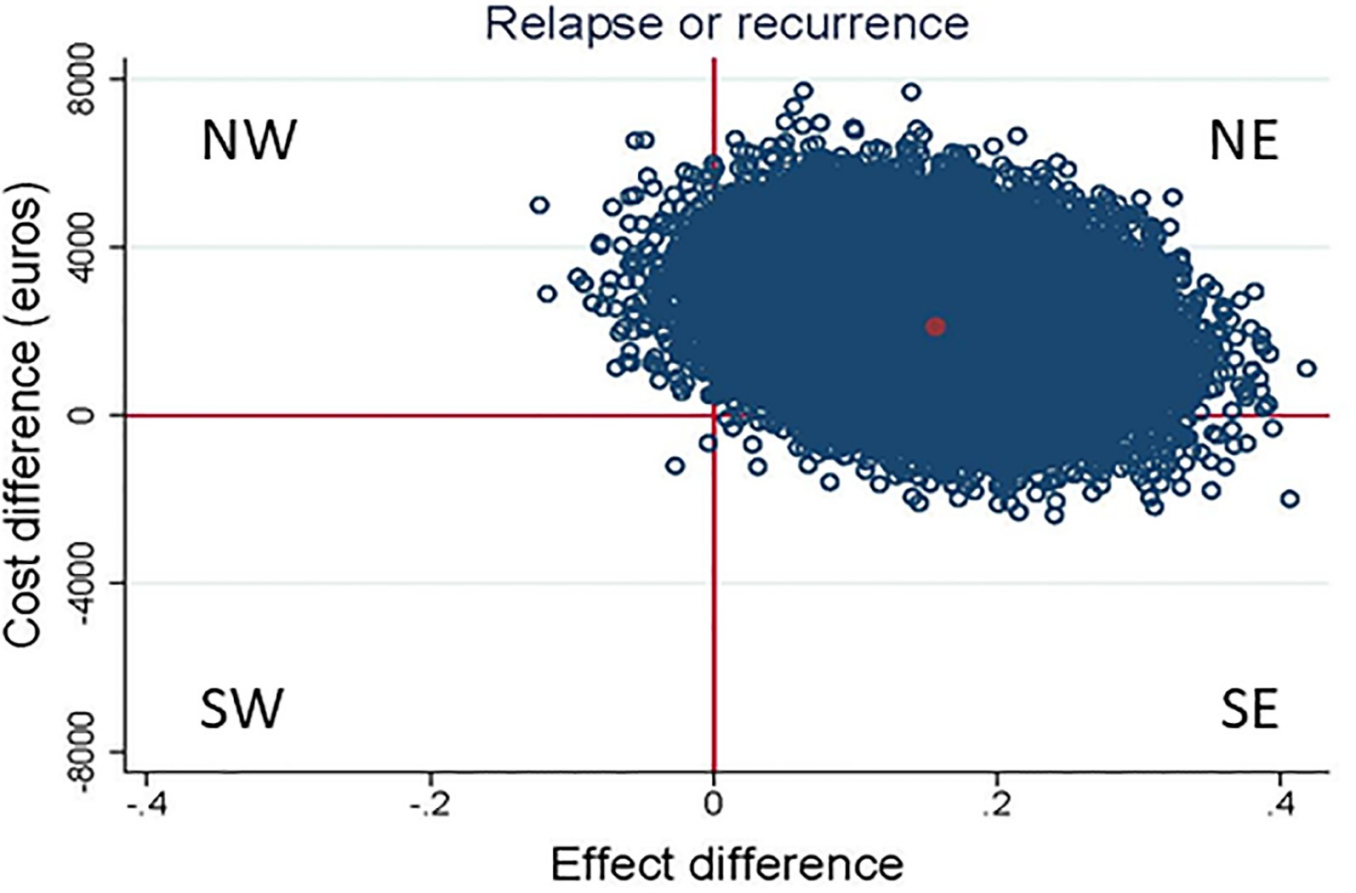
Scatter plot of estimated relapse of recurrence of incremental costs and incremental effects of S-PCT vs TAU obtained by bootstrap re-sampling. NE; more expensive, more effective, SE; less expensive, more effective, SW; less expensive, less effective, NW; more expensive, less effective.

**Fig 3:**
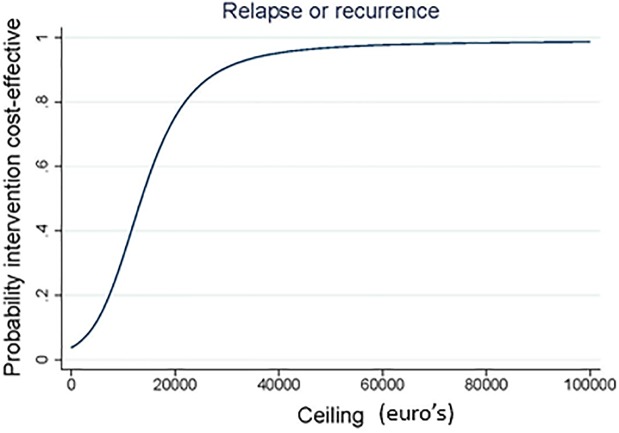
Cost-effectiveness acceptability curve for relapse of recurrence showing the probability that S-PCT is cost-effective compared to TAU over a range of values for the maximum acceptable ceiling.

The results of the CEA are presented in Table 3, Figs 2 and 3. The ICER for depressive relapse or recurrence was 13,515, indicating that €13,515 should be invested to prevent 1 relapse or recurrence in the S-PCT group in comparison with the TAU group. The CE-plane shows that 96% of the bootstrapped cost-effect pairs were located in the NE quadrant (more effective and more expensive). The accompanying CEAC showed that the probability that the intervention was considered cost-effective was 4% if WTP is 0 €/ relapse or recurrence prevented, 80% if WTP is 22,000 €/ relapse or recurrence prevented, and 95% if WTP is 39,500 €/ relapse or recurrence prevented.

**Table 3:** Differences in relapse or recurrence and costs over 12 months between S-PCT and TAU; ICER, CE-planes quadrants, and acceptability.

Analysis Outcome; relapse or recurrence	Costs Δ^a^ (95% CI)	Effect Δ^b^ (95% CI)	ICER	Cost-effectiveness plane	Cost-effectiveness plane	Cost-effectiveness plane	Cost-effectiveness plane	Probability that S-PCT is cost-effective compared to TAU at WTP of €0	Minimum willingness to pay for the intervention to be cost-effective compared to TAU at given probabilities	Minimum willingness to pay for the intervention to be cost-effective compared to TAU at given probabilities
				NE	SE	SW	NW		0.80 (80%)	0.95 (95%)
**ITT analysis (societal perspective)**	2,114 (-112;4261)	0.15 (0.03;0.28)	13,515	96%	3%	0%	1%	4%	€22,000	€39,500
**Per-protocol analysis (societal perspective)**	1,808 (-495;4025)	0.17 (0.03;0.30)	10,602	94%	5%	0%	1%	6%	€17,500	€30,500
**ITT analysis (healthcare system perspective)**	1,107 (75;2322)	0.15 (0.03;0.28)	7,079	97%	3%	0%	1%	4%	€11,500	€21,500

CEA, cost-effectiveness analysis; CI, confidence interval; CUA, cost-utility analysis; ICER, incremental cost effectiveness ratio; ITT, intention-to-treat; NE, north east; NW, north west; SE, south east; SW, south west; TAU, treatment-as-usual;WTP, willingness to pay

^a^ Regression analysis; adjusted for baseline costs

^b^ Poisson regression analysis, adjusted for depressive symptoms

### Cost-utility; primary results

**Fig 4:**
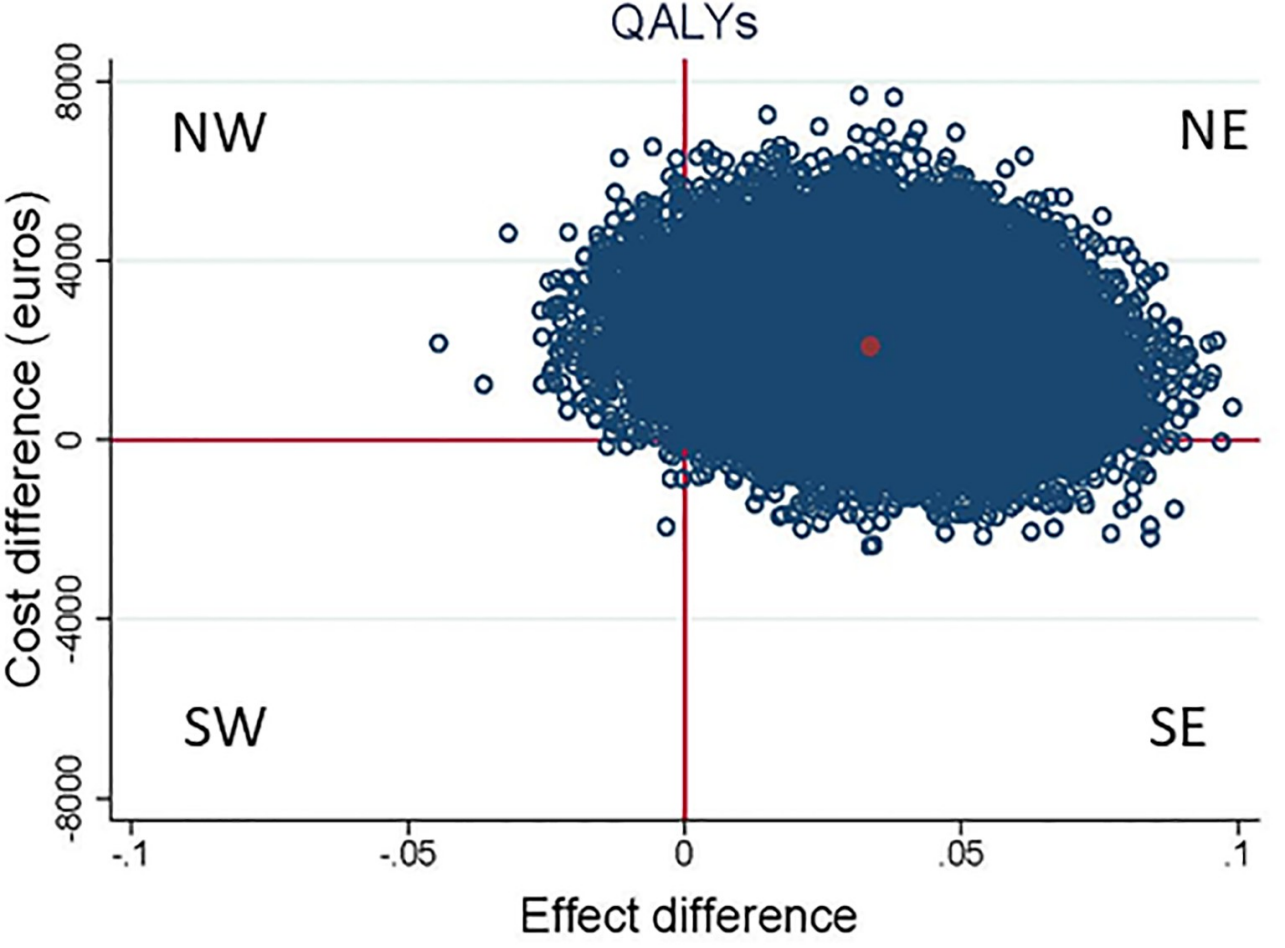
Scatter plot of estimated QALY of incremental costs and incremental effects of S-PCT vs TAU obtained by bootstrap re-sampling. NE; more expensive, more effective, SE; less expensive, more effective, SW; less expensive, less effective, NW; more expensive, less effective.

**Fig 5:**
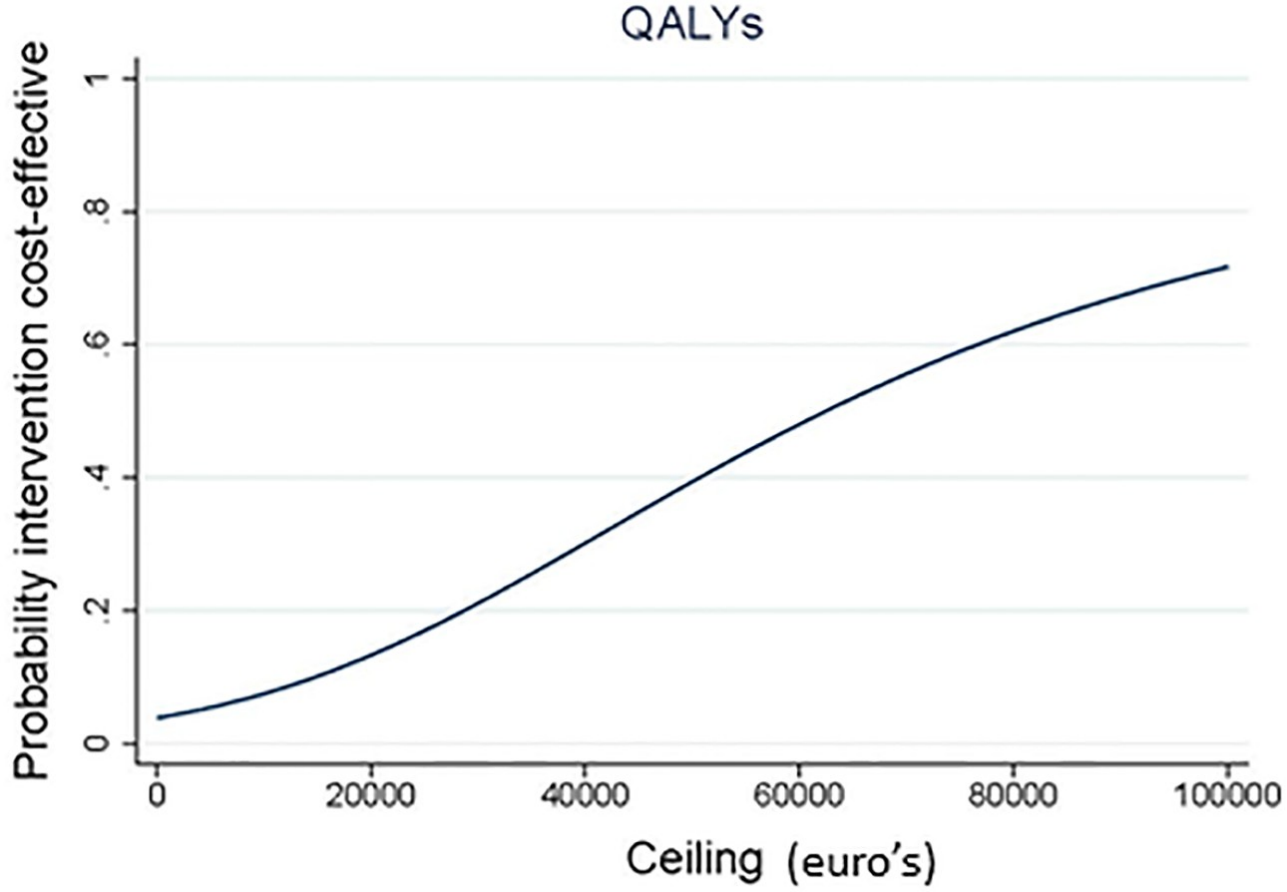
Cost-effectiveness acceptability curve for QALY showing the probability that S-PCT is cost-effective compared to TAU over a range of values for the maximum acceptable ceiling.

The results of the CUA are presented in Table 4, Figs 4 and 5. The ICER for QALYs was 63,051 indicating that €63,051 should be invested to gain 1 QALY in the S-PCT group as compared to the TAU group. The CE-plane showed that 96% of QALY cost-effect pairs were located in the NE quadrant (more effective and more expensive). The CEAC showed that the probability that the intervention was considered cost-effective was 13% if WTP is 20.000 €/QALY gained and that this slowly increased to 21% if WTP is 30,000 €/QALY gained.

**Table 4:** Differences in QALYs and costs over 12 months between S-PCT and TAU; ICER, CE-planes quadrants, and acceptability.

Analysis Outcome; QUALY	Costs Δ^a^ (95% CI)	Effect Δ^b^ (95% CI)	ICER	Cost-effectiveness plane	Cost-effectiveness plane	Cost-effectiveness plane	Cost-effectiveness plane	Probability that S-PCT is cost-effective compared to TAU at WTP of €0	Probability that S-PCT is cost- effective compared to TAU at given WTP	Probability that S-PCT is cost- effective compared to TAU at given WTP
				NE	SE	SW	NW	WTP €0	WTP €20.000	WTP €30.000
**ITT analysis (societal perspective)**	2,114 (-112;4261)	0.03 (0.0006;0.07)	63,051	95%	3%	0%	2%	4%	13%	21%
**Per-protocol analysis (societal perspective)**	1,808 (-495;4025)	0.04 (0.008:0.08)	41,952	94%	5%	0%	1%	6%	23%	36%
**ITT analysis (healthcare system perspective)**	1,107 (75;2322)	0.03 (0.0006;0.07)	33,025	95%	3%	0%	2%	3%	28%	46%

CEA, cost-effectiveness analysis; CI, confidence interval; CUA, cost-utility analysis; ICER, incremental cost effectiveness ratio; ITT, intention-to-treat; NE, north east; NW, north west; QALY, quality-adjusted-life-year; SE, south east; SW, south west; TAU, treatment-as-usual; WTP, willingness-to-pay

^a^ Adjusted for baseline costs

^b^ Linear mixed models, adjusted for depressive symptoms

### Sensitivity analyses

The results of the sensitivity-analyses are presented in Table 3 (relapse or recurrence), and Table 4 (QALY). The analysis from healthcare perspective showed that adjusted costs in the S-PCT group were statistically significantly lower than in the TAU group (mean difference €1,107, 95%CI 75;2322). The effect differences are the same as in the ITT analysis from the societal perspective. The ICER for depressive relapse or recurrence was 7,079 indicating that €7,079 should be invested to prevent 1 relapse or recurrence in the S-PCT group in comparison with the TAU group. The majority of the cost-effect pairs were again located in the NE quadrant (97%). The accompanying CEAC showed that the probability that the intervention was cost-effective was 4% if WTP is 0 €/ relapse or recurrence prevented, 80% if WTP is 11,500 €/ relapse or recurrence prevented, and 95% if WTP is 21,500 €/ relapse or recurrence prevented. From a healthcare perspective, the ICER for QALYs was 33,025, indicating that €33,025 should be invested to gain 1 QALY in the S-PCT group as compared to the TAU group. The probability that S-PCT was considered cost-effective in comparison with TAU for QALYs at WTP thresholds of €20.000 and €30.000 were 28% and 46%, respectively.

A per protocol (PP) analysis with only those participants who completed at least 80% (5 modules) of the intervention (81%; 101 out of 124 participants), showed a mean difference in costs between the S-PCT group and the TAU group of €1,808 (95%CI €-495;4025). S-PCT decreased relapse or recurrence by 17% (95%CI 3;30) and gained 0.04 QALY (95%CI 0.008;0.08) compared to the TAU group. The ICER for depressive relapse or recurrence was 10,602. Again, the cost-effect pairs were mostly located in the NE quadrant of the CE plane (94%). The accompanying CEAC showed that the probability that the intervention was cost-effective was 6% if WTP is 0 €/relapse or recurrence prevented, 80% if WTP is 17,500 €/relapse or recurrence prevented, and 95% if WTP is 30,500 €/relapse or recurrence prevented. In the PP analysis, the ICER for QALYs was 41,952. The probability that S-PCT was considered cost-effective in comparison with TAU for QALYs at WTP thresholds of €20,000 and €30,000 were 23% and 36%, respectively.

## Discussion

### Main findings

We evaluated whether treatment-as-usual (TAU) augmented with a supported self-help Preventive Cognitive Therapy (S-PCT) in primary care was cost-effective in comparison with TAU alone for patients with recurrent depression. In the S-PCT group, statistically significantly fewer patients experienced a relapse or recurrence of depression than in the TAU group. For QALYs, a statistically significant difference between the two conditions was also observed, in favor of the S-PCT group. However, mean societal costs adjusted for baseline costs were higher in the S-PCT group than in the TAU group over 12 months, though not statistically significantly. Willingness-to-pay values should be quite high to reach an acceptable probability that S-PCT is considered cost-effective in comparison with TAU (80% at a WTP of 22,000 €/relapse or recurrence prevented). For the commonly accepted WTP value of 20,000 €/QALY gained, the probability that S-PCT was cost-effective in comparison with TAU was 13%. Sensitivity analyses showed similar results.

### Interpretation and explanation of main findings

The main contributors to the difference in mean societal costs were the costs due to secondary medical care (mental health care not included) and due to lost productivity. It is unclear how these cost differences can be explained as there were no relevant differences in baseline characteristics, there were no clear outliers for costs and there was no association between costs and relapse or recurrence in the S-PCT group.

Previous research shows that interventions (usually CBT) aimed at the prevention of the *onset* of MDD in patients with sub-threshold depression (depressive symptoms but insufficient to warrant MDD), generally show good value for money [51–55]. However, the few economic evaluations of preventive interventions to reduce the risk of *relapse or recurrence* of MDD, show mixed results. Stant et al. [56] found that TAU enriched with CBT might be cost-effective in preventing relapses in primary care patients with depression compared to TAU alone. Psychodynamic counselling for relapse prevention proved to be cost-effective in comparison with usual care [57]. The study by Kuyken et al. [58] is the most recently published cost-effectiveness analysis, indicating that MBCT plus the tapering of ADM is not cost-effective in comparison with maintenance ADM in terms of either relapse or recurrence or QALYs in patients with a history of three or more depressive episodes and on a therapeutic dose of maintenance antidepressant drugs medication at baseline. A closer look at their costs reveals that the mean societal costs over 24 months per participant in the intervention group and control group in the study by Kuyken et al. (₤3,204/€4,415 and ₤2,754/€3,795 respectively) were much lower than the mean societal costs over 12 months per participant in our study (€7,897 and €5,065, respectively). Kuyken et al. used other ways of measuring productivity losses and healthcare costs, which might explain some of this difference. However, for example, their mean number of fulltime days off work was half the number of days in our study. An explanation for this difference in days off work is lacking.

### Strengths and limitations

This study has several strengths. To minimise recall bias, costs over the 12-month follow-up were assessed with questionnaires at 3,6,9 and 12 months. Furthermore, our measurement of depression was based on a well-validated and reliable structured clinical interview (Structured Clinical Interview for DSM-IV Disorders-1)[25]. A further strength of this study is that the trial participants achieved remission or recovery on antidepressants, other psychotherapies, psychiatric help, counselling, or no treatment at all, as is typically the case in clinical practice. Moreover, there were no restrictions in the use of medication at entry to the study. This was done to maximise the external validity of the study, resulting in high generalizability of the findings.

A limitation of our study is, as is common in studies on psychological interventions, that it was not possible to blind participants to the condition to which they were assigned. Also, the follow-period of 1 year might have been too short to capture all the changes in effects and costs due to the intervention. Time to recurrence might exceed 12 months, possibly implying that we have missed the interventions’ and TAUs’ impact on later recurrences and associated health-economic consequences (such as productivity and health care use). Finally, we used the EQ-5D to detect differences in health care status and to compare the outcomes with other studies. However, the EQ-5D has shown to be less responsive and needs larger patient samples to detect meaningful differences in patients with depression [59]. Though our sample includes remitted patients, this limitation may have influenced the results and the clinical effects may have been larger with another instrument.

### Conclusions and future research

Our study shows that the larger effects of S-PCT are associated with higher costs as compared to TAU. A WTP of 22,000 €/relapse or recurrence prevented to reach a 80% probability that S-PCT is cost-effective in comparison with TAU is expected to be too high for decision makers. We recommend an extended follow-up in future studies and a more in-depth evaluation of costs. Furthermore, we recommend offering S-PCT directly after treatment in the acute phase [60]. In addition, it is crucial to study what works for whom.

## Supporting information

S1 Protocol (DOC)

S1 Dataset (SAV)

S1 Checklist (DOC)
