# Supplementary material for: A supported self-help for recurrent depression in primary care; An economic evaluation alongside a multi-center randomised controlled trial
Source: PLoS One. 2018 Dec 19;13(12):e0208570. doi: 10.1371/journal.pone.0208570 (PMC6300246; doi:10.1371/journal.pone.0208570)
Supplement: S1 Protocol — (DOC) [file pone.0208570.s001.doc]

**Nurse-led self-help for recurrent depression versus usual care in the primary care setting: pragmatic randomised trial**

**and economic evaluation**

**(August - 2011)**

**Project number EMGO** OZ 06072055 / WC 2009-080

**ZonMw project number** 80-82310-97-11087

**ABR Form Number** 37685

**METC number** NL37685.029.11

**Trial registration number** xxx

**EudraCT number** n/a

**‘Nurse-led self-help for recurrent depression versus usual care in the primary care setting: pragmatic randomised trial and economic evaluation’**

| **Protocol ID** | **OZ06072055** |
| --- | --- |
| **Short title** | **Self- help for recurrent depression in primary care** |
| **Version** | **1** |
| **Date** | **august 2011** |
| **Coordinating investigator/project leader** | ***Dr. HWJ van Marwijk,* GP, associate professor of general practice, VU medical centre** Van der Boechorststraat 7  1081 BT Amsterdam The Netherlands T: + 31 20 4449368 T: + 6 28551084 E: hwj.vanmarwijk@vumc.nl  ***Prof dr F Smit,*** Professor of Evidence-Based Public Mental Health, **VU medical centre Van der Boechorststraat 1 1081 BT, Amsterdam The Netherlands  T: +31 20 5988847  M +31 6 29735268  E: f.smit@psy.vu.nl** |
| **Principal investigator(s) (in Dutch: hoofdonderzoeker/uitvoerder)**  ***Multicenter research: per site*** | ***Drs. KEM Leliefeld****, MD,* VU medical centre Van der Boechorststraat 7  1081 BT Amsterdam The Netherlands T: + 31 20 444 8395  M: + 31 6 24762533 E: k.leliefeld@vumc.nl |
| **Sponsor (in Dutch: verrichter/opdrachtgever)** | **ZonMw** |
| **Independent physician(s)** | ***Otto Maarsingh, GP***  EMGO Institute **VUmc** P.O. Box 7057  1007 MB Amsterdam The Netherlands 020 - 444 8167 ( Monday, Thursday ) 020 - 444 1400 ( Wednesday, Friday ) |
| **Laboratory sites <*if applicable*>** | ***n/a*** |
| **Pharmacy <*if applicable*>** | ***n/a*** |
| **Projectmembers** | **Prof dr T. van Balkom (psychiatry)**  T + 31 20 7884555  E t.vanbalkom@ggzingeest.nl |
| **Co-promotor** | **Dr A. van Schaik**  T + 31 20 7884535  E a.vanschaik@ggzingeest.nl |
|  | **Dr C. Bockting (psychology)**  T +31 50 3636479  E [c.l.h.bockting@rug.nl](mailto:c.l.h.bockting@rug.nl) |
| **Co-promotor** | **Dr H.W.J. van Marwijk**  T: + 31 20 4449368  E: hwj.vanmarwijk@vumc.nl |
| **Promotor** | **Prof dr F. Smit (psychology)**  **T: +31 20 5988847  E: f.smit@psy.vu.nl** |
|  | **Dr J. Bosmans (KEA-expert)**  T + 31 20 5982843  E j.e.bosmans@ vu.nl |
|  | **Dr V. van der Feltz**  T +31 30 297111  E cfeltz@trimbos.nl |
| **Promotor** | **Prof dr H.E. van der Horst (PCP)**  T + 31 20 4449311  E he.vanderhorst@vumc.nl |
|  | **Dr B. Terluin (PCP)**  T +31 20 4449515  E b.terluin@vumc.nl |
|  | **Prof Dr J. Savelkoul**  T +31 20 4444444  j.savelkoul@vumc.nl |

**PROTOCOL SIGNATURE SHEET**

| **Name** | **Signature** | **Date** |
| --- | --- | --- |
| **Head of Department:**  **Prof dr H.E. van der Horst (PCP)**  T + 31 20 4449311  E he.vanderhorst@vumc.nl |  | **August 2011** |
| **Coordinating Investigator / Project leader / Principal Investigator:**  ***Dr. HWJ van Marwijk,***  **GP, associate professor of general practice, VU medical centre** Van der Boechorststraat 7  1081 BT Amsterdam The Netherlands T: + 31 20 4449368 E: [HWJ.vanmarwijk@vumc.nl](mailto:HWJ.vanmarwijk@vumc.nl)  ***Drs. K.E.M. Leliefeld, MD***  PhD candidate, VU medical centre Van der Boechorststraat 7  1081 BT Amsterdam The Netherlands T: + 31 20 4448395  E: k.leliefeld[@vumc.nl](mailto:HWJ.vanmarwijk@vumc.nl) |  | **August 2011**  **August 2011** |

**TABLE OF CONTENTS**

1. INTRODUCTION AND RATIONALE 11

2. OBJECTIVES [14](#__RefHeading___Toc295377454)

3. STUDY DESIGN 17

4. STUDY POPULATION ……………………………………………………………………….....17

4.1 Population (base) [18](#__RefHeading___Toc295377456)

4.2 Inclusion criteria [18](#__RefHeading___Toc295377457)

4.3 Exclusion criteria [19](#__RefHeading___Toc295377458)

4.4 Sample size calculation [19](#__RefHeading___Toc295377459)

5. TREATMENT OF SUBJECTS [20](#__RefHeading___Toc295377460)

5.1 Investigational product / treatment 20

5.2 Use of co-intervention (if applicable) [21](#__RefHeading___Toc295377465)

5.3 Escape medication (if applicable) [21](#__RefHeading___Toc295377466)

6. INVESTIGATIONAL MEDICINAL PRODUCT [21](#__RefHeading___Toc295377467)

7. METHODS [22](#__RefHeading___Toc295377468)

7.1 Study parameters/endpoints [22](#__RefHeading___Toc295377469)

7.1.1. Main study parameter/endpoint [22](#__RefHeading___Toc295377470)

7.1.2. Secondary study parameters/endpoints [22](#__RefHeading___Toc295377473)

7.1.3. Other study parameters (if applicable) [22](#__RefHeading___Toc295377480)

7.2 Randomisation, blinding and treatment allocation [22](#__RefHeading___Toc295377481)

7.3 Study procedures [22](#__RefHeading___Toc295377482)

7.4 Withdrawal of individual subjects [24](#__RefHeading___Toc295377483)

7.4.1 Specific criteria for withdrawal [24](#__RefHeading___Toc295377484)

7.5 Replacement of individual subjects after withdrawal [24](#__RefHeading___Toc295377485)

7.6 Follow-up of subjects withdrawn from treatment [24](#__RefHeading___Toc295377486)

7.7 Premature termination of the study [25](#__RefHeading___Toc295377487)

8. SAFETY REPORTING [26](#__RefHeading___Toc295377488)

8.1 Section 10 WMO event [26](#__RefHeading___Toc295377489)

8.2 Adverse and serious adverse events [26](#__RefHeading___Toc295377490)

8.2.1. Suspected unexpected serious adverse reactions (SUSAR) [27](#__RefHeading___Toc295377491)

8.2.2. Annual safety report [27](#__RefHeading___Toc295377492)

8.3 Follow-up of adverse events [27](#__RefHeading___Toc295377493)

8.4 Data Safety Monitoring Board (DSMB) [27](#__RefHeading___Toc295377494)

9*.* STATISTICAL ANALYSES [28](#__RefHeading___Toc295377495)

9.1 Descriptive statistics [28](#__RefHeading___Toc295377496)

9.2 Analysis of primary clinical outcome………………………………………………………..28

9.3 Economic evaluation…………………………………………………………………………28

9.4 Analyses of secondary clinical outcomes 29

9.5 Analyses of secondary economic outcome…………………………………………….....29

9.6 Interim analysis (not applicable)……………………………………………………………30

10. ETHICAL CONSIDERATIONS [31](#__RefHeading___Toc295377500)

10.1 Regulation statement [31](#__RefHeading___Toc295377501)

10.2 Recruitment and consent [31](#__RefHeading___Toc295377502)

10.3 Objection by minors or incapacitated subjects (if applicable) [31](#__RefHeading___Toc295377503)

10.4 Benefits and risks assessment, group relatedness [31](#__RefHeading___Toc295377504)

10.5 Compensation for injury [31](#__RefHeading___Toc295377505)

10.6 Incentives (if applicable) [31](#__RefHeading___Toc295377506)

11. ADMINISTRATIVE ASPECTS AND PUBLICATION [32](#__RefHeading___Toc295377507)

11.1 Handling and storage of data and documents [32](#__RefHeading___Toc295377508)

11.2 Amendments [32](#__RefHeading___Toc295377509)

11.3 Annual progress report [32](#__RefHeading___Toc295377510)

11.4 End of study report [32](#__RefHeading___Toc295377511)

11.5 Public disclosure and publication policy [32](#__RefHeading___Toc295377512)

12. REFERENCES [33](#__RefHeading___Toc295377513)

**LIST OF ABBREVIATIONS AND RELEVANT DEFINITIONS**

| **ABR** | ABR form, General Assessment and Registration form, is the application form that is required for submission to the accredited Ethics Committee (In Dutch, ABR = Algemene Beoordeling en Registratie) |
| --- | --- |
| **AE** | Adverse Event |
| **AR** | Adverse Reaction |
| **CA** | Competent Authority |
| **CCMO** | Central Committee on Research Involving Human Subjects; in Dutch: Centrale Commissie Mensgebonden Onderzoek |
| **CV** | Curriculum Vitae |
| **DSMB** | Data Safety Monitoring Board |
| **EU** | European Union |
| **EudraCT** | European drug regulatory affairs Clinical Trials |
| **GCP** | Good Clinical Practice |
| **IB** | Investigator’s Brochure |
| **IC** | Informed Consent |
| **IMP** | Investigational Medicinal Product |
| **IMPD** | Investigational Medicinal Product Dossier |
| **METC** | Medical research ethics committee (MREC); in Dutch: medisch ethische toetsing commissie (METC) |
| **(S)AE** | (Serious) Adverse Event |
| **SPC** | Summary of Product Characteristics (in Dutch: officiële productinfomatie IB1-tekst) |
| **Sponsor** | The sponsor is the party that commissions the organisation or performance of the research, for example a pharmaceutical  company, academic hospital, scientific organisation or investigator. A party that provides funding for a study but does not commission it is not regarded as the sponsor, but referred to as a subsidising party. |
| **SUSAR** | Suspected Unexpected Serious Adverse Reaction |
| **Wbp** | Personal Data Protection Act (in Dutch: Wet Bescherming Persoonsgevens) |
| **WMO** | Medical Research Involving Human Subjects Act (in Dutch: Wet Medisch-wetenschappelijk Onderzoek met Mensen |

**SUMMARY**

***Rationale*:** Major Depressive Disorder (MDD) makes a major contribution to disability and healthcare costs and tends to run a recurrent course. Because the long-term outcome of depression is often unfavourable, there is an urgent need for a (cost-) effective psychotherapy, aimed at the prevention of depression in remitted patients, which is readily accessible at the primary care level. Preventive cognitive therapy (PCT) has already proven to be effective in the prevention of recurrence in group sessions. For both cost-effectiveness and pragmatic reasons, the PARADE study applies PCT in the primary care as a nurse-led self-help. It is hypothesized that adding nurse-led self-help to usual care is clinically superior to care as usual alone for preventing recurrence in recurrent depressive disorder. In addition, it is expected that the intervention dominates the comparator condition in terms of cost-effectiveness.

***Objective***: The primary objective of this study is to evaluate whether nurse-led, cognitive therapy based self-help is (cost-) effective for patients with recurrent MDD in primary care in comparison with usual care. Furthermore this study examines whether nurse-led self-help for patients with recurrent MDD versus usual care alone A) reduces health care utilisation, B) is effective in reducing co-morbid distress, anxiety and somatisation, C) is satisfying according to patients and D) is (cost)-effective in certain subgroups of patients.

***Study design*:** This study is a pragmatic randomised controlled trial among primary care patients with remitted MDD. Patients are randomly assigned to a ‘nurse-led self-help therapy plus usual care’ (134 patients) or ‘usual care alone’ (134 patients). Stratification variables are the number of previous episodes and the type of treatment as usual received during the last episode. It is not possible to blind patients, healthcare providers and main researcher to the intervention in this study due to the nature of the intervention.

***Study population:*** The study population consists of currently remitted patients (age 18-65) with two or more previous episodes of MDD. Participants who have a current mania or a history of bipolar disease, any current organic brain disorder, psychotic disorder or participants with severe sensory disabilities are excluded from participation. Eligible patients are found through a search in the database in the participating primary care practices.

***Intervention***: The intervention consists of a nurse-led self-help based on Preventive Cognitive Therapy (PCT). Patients are offered a detailed treatment manual of the therapy with literature, backgrounds and assignments, and have weekly telephone contact with a nurse. The therapy consists of eight sessions with a fixed structure. Patients in the control condition receive care as usual.

***Main study parameters/endpoints*:** Primary outcome is the cumulative rate of recurrences of depression meeting DSM-IV criteria, assessed with the Structured Clinical Interview for DSM-IV at baseline and at 3,6,9,12 and 15 months follow-up. For the economic evaluation, quality of life is measured using the EuroQol and 12-item Short Form Health Survey (SF-12). Costs are measured from a societal perspective using the TIC-P. Moderator analyses are conducted for demographic characteristics, number of previous depressive episodes, age of onset of first depression, self-efficacy in preventing depression, satisfaction with PCT, pain and fatigue in order to indentify subgroups that show particularly good response to the intervention.

***Nature and extent of the burden and risks associated with participation, benefit and group relatedness*:** Benefits regarding participation in this trial are the effective preventive cognitive therapy that is offered to patients in a new setting and a regular check for depressive symptoms. Burden associated with participation are the questionnaires that patients must fill in at 3,6,9,12 and 15 months follow-up and the time to complete the assignments of the self-help (approximately 1,5 hours a week). Risks in this trial are very low because usual care, including the use of AD, is permitted in both arms.

# 1. INTRODUCTION AND RATIONALE

**1.1 Introduction**

Major Depressive Disorder (MDD) is estimated by the World Health Organization to be a leading cause for loss of disability-adjusted life years and makes a major contribution to disability and healthcare costs^1;2^. MDD tends to run a relapsing and recurrent course. Accordingly, interventions to reduce the disabling effects of depression should be aimed at the prevention of depressive relapses/recurrences ^3^. Henceforth, ‘relapse/recurrence’ is captured in the term ‘recurrence’.

Both psychological and pharmacologic therapies are effective in the long-term treatment of patients with depressive disorders and each has its own merits. For several years, maintenance treatment with antidepressants (AD) has been the leading strategy to prevent recurrence in patients with recurrent MDD. However, the quality of the evidence to support such prolonged treatment is poor^4-6^. A majority of the patients are not willing to take AD for a long period of time^7;8^. Therefore, adherence in AD users is estimated at only 50% at best ^7-9^. Patients’ protection from recurrence ceases on discontinuation of AD ^6^ and patients might develop resistance against the prophylactic properties of AD^10^. Furthermore, for some patients, AD may be contra-indicated because of somatic illness or side effects. Last but not least, the optimal duration of the maintenance treatment has not been studied. Particularly in primary care, recommendations on maintenance treatment with antidepressants cannot be considered evidence-based ^11^.

Only more recently attention has turned to psychotherapy in preventing recurrence in recovered depressed patients. Many depressed patients prefer psychological treatments over drugs ^12^. According to a review by Hollon et al (2010), Cognitive Behavioural Therapy (CBT) is efficacious (…) in the maintenance treatment of recurrent MDD ^13^. A meta-analysis by Vittengl et al of 28 studies including 1,880 adults, demonstrated that among acute-phase treatment responders, CBT substantially reduced the number of recurrences compared to assessment only at the end of continuation treatment. Preventive cognitive therapy (PCT), an adaptation of the CBT protocol is also effective in the prevention of recurrence in major depression^14^. This preventive cognitive therapy differs from other cognitive therapies as it mainly focuses on so-called ‘restricting rules of living’. It is assumed that these ‘restricting rules’ are still existent while being remitted and therefore cause vulnerability for recurrence. In this therapy patients are learned to recognise these rules and how to change them. Furthermore, the therapy focuses on identifying and changing dysfunctional attitudes, and especially enhances specific memories of positive experience by keeping a diary of positive experiences and formulating specific recurrence prevention strategies^15^.

Most of the cognitive therapies take place at the specialist level, drawing on scarce resources. The vast majority of depressed patients visit - and are treated by their primary care physician (PCP) first however^16^. In the Netherlands, psychological therapies have relatively high costs and reimbursement for psychotherapy in primary care is limited. Besides, evidence based psychotherapies are less readily accessible in primary care^12^.

Because the long-term outcome of major depression is often unfavourable, there is an urgent need for a (cost-) effective psychotherapy, aimed at the prevention of depression in remitted patients, which is readily accessible at the primary care level. The need for psychological intervention might be especially pronounced in patients who are adverse to using antidepressants (either because they have shown no treatment response to pharmaceutical intervention in the past, or have not been very adherent to pharmaceutical intervention) and in patients who are at high risk for recurrence.

Several risk factors for recurrent depression have been defined ^17^ including premature anti-depressant withdrawal, socio-demographic factors such as age and gender, longer duration of individual episodes and poor symptom control during continuation therapy. Younger age of onset of first depressive episode, low socioeconomic status, low self-efficacy for managing depression and symptoms of pain and/or fatigue also act as risk factors for recurrent depression.

Besides, several subgroup analyses, based on stratified ^18,19^ and non-stratified ^15^ subsamples, suggest that PCT is more effective in ‘high risk’ patients meaning patients with a history of at least three episodes on a life time basis. Recall-bias often hampers assessment of the number of previous episodes on a life-time basis. Therefore it is better to select patients on the number of episodes during a shorter time frame of say, 2 episodes in the last five years.

Only few studies have focused on psychological interventions aimed at the prevention of recurrences in primary care patients with depression ^20^.

**1.2 Rationale**

Preventive cognitive therapy (PCT) has proved to be effective in preventing recurrent depression, and seems to very suitable to deliver in primary care because of its very structured design. ‘Bibliotherapy’ or self-help intervention is the most accessible form of psychological intervention for recurrent depressed primary care patients and is defined as the use of written, audio, or e-learning materials to provide therapeutic support in mental health service. Research indicates that cognitive bibliotherapy, has a moderate to large effect in reducing symptoms of depression and anxiety ^21-24^.

Self-help interventions can be purely self-administered or can be used as part of a ‘guided’ therapist-led intervention. Studies show that self-help therapies may be sufficient for several anxiety and depressive disorders but that some form of support may be essential for enhancing compliance with the ultimate aim of improving treatment outcomes ^24-27^. Some form of guidance is likely to be important because the motivation in remitted patients to actively participate in self-help might be relatively low and because the therapy might be difficult at certain stages. Hence, some contact between patients and therapist is generally speaking a good idea. A disadvantage of guided self-help interventions is that they may impose a time burden on PCP’s. Also, PCP’s may lack the necessary training to administer these interventions. Recent studies have shown that paraprofessionals, like nurses or prevention-workers, can be successfully trained to administer forms of CBT-based self-help interventions, and thus may complement the regular work of the PCP’s ^28;29^.

In this study, contact with the nurse is mainly supportive or facilitative and includes no active therapeutic engagement.

From 2008 onwards, ambulatory psychiatric care is included in Dutch healthcare insurance and PCP’s can employ a mental health nurse in their practices for four hours a week per PCP. For both cost-effectiveness and pragmatic reasons it is therefore attractive to let a nurse play a pivotal and facilitating role.

Therefore, this study evaluates a nurse-led, cognitive therapy based self-help, for patients at high risk for recurrent depression in primary care.

**1.3 Other studies**

According to the clinicaltrials.gov website of the U.S. National Institutes of Health (search

terms: ‘prevention’, ‘self-help’, ‘depression’, primary care’, ‘preventive cognitive therapy’, ‘nurse-led’), no trials are or have been conducted that resemble ours. To the best of our knowledge, there are no studies underway that are similar to the proposed trial.

#

# 2. OBJECTIVES

**Primary Objective:**

To study whether nurse-led self-help is (cost-) effective for persons with recurrent MDD in primary care in comparison with usual care. Effectiveness is defined as significantly less SCID/DSM-V recurrences of MDD in one year follow-up compared to usual care. Cost-effectiveness is evaluated from a societal perspective meaning that the costs of the intervention, other health care utilization, patients' out-of-pocket costs and costs due to productivity losses will be included in the economic evaluation.

It is hypothesized that adding nurse-led self-help to usual care is clinically superior to care as usual alone for preventing recurrence in recurrent depressive disorder. In addition, it is expected that the intervention dominates the comparator condition in terms of (cost-) effectiveness.

**Secondary Objective(s):**

To study whether nurse-led self-help for patients with recurrent MDD versus usual care alone:

A) reduces health care utilisation

B) is effective in reducing co-morbid distress, anxiety and somatisation

C) is satisfying according to patients

D) is (cost)-effective in certain subgroups of patients, particularly related to (i.e. modified by):

- the number of previous episodes

- type of treatment for the last recurrence (AD, psychological intervention, nothing etc).

- the severity of residual depressive symptoms in the remitted phase

- age of onset of the first depressive episode,

- social economic status (education)

- self-efficacy for managing depression

- symptoms of pain and/or fatigue

- long duration of individual episodes

- socio-demographic factors such as age and gender

- pattern use of antidepressants

The secondary objectives lead to the hypotheses below about effect modification.

*RE: Reduction in health care utilisation*

The substantial economic burden of depression is partly due to an increased health care utilisation. Evidence shows that health care costs of depressed patients are higher compared with matched controls even after adjusting for co-morbidity^30^. This is not only true for mental but also for general medical care utilization. Therefore, it is hypothesized that by preventing recurrences through this intervention, health care utilisation and related costs in these patients will also be reduced.

*RE: B (cost-) effective in reducing co-morbid levels of psychopathology*

Psychiatric comorbidity in depression is associated with greater symptom severity, severer functional impairments and a more chronic course of the disease. Comorbid psychopathology in depressed patients is common and often involves anxiety and somatisation.

Anxiety consists of irrational and diffuse fears and avoidance behaviour. Somatisation consists of a range of psychosomatic symptoms like headache, muscle pains, cardiovascular and gastrointestinal symptoms^31^. It is hypothesized that with the reduction of (risk for) recurrences, the co-morbid level of anxiety and/or somatisation will also decrease.

Unlike anxiety and somatisation, distress is *always* part of the symptomatology of a depressive disorder. Distress contains nonspecific symptoms of psychopathology, ranging from worrying and irritability to fatigue and demoralization. It is hypothesized that symptoms of distress in remitted patients who are in the nurse-led self-help group will decrease due to a lesser likelihood of recurrence.

*RE: C is satisfying according to patients*

Patients’ satisfaction with their treatment is an important predictor of the overall clinical outcome and is a widely used measurement for the evaluation of medical care. In this trial the nurse-led self-help is a new way of delivering a preventive cognitive therapy and satisfaction therefore is unknown. The hypothesis is that patients with higher scores on satisfaction with this format experience a better overall outcome in terms of prevention of recurrences.

*RE: D is (cost-) effective in certain subgroups of patients*

A number of baseline characteristics of the patients that are prognostically relevant for treatment response can be identified. It is hypothesized that patients at higher risk for recurrence benefit the most from this intervention, particularly patients with three to five, or more, prior episodes^15;18;19^. Besides, patients that already underwent a psychological intervention have the benefit of possible long-term protection for recurrence. Residual depressive symptoms, occurring in many remitted patients, are associated with a much increased risk for recurrence, particularly in the first year ^32^. Therefore it is hypothesized that with the presence of residual symptoms in remitted patients the risk for recurrence subsequently increases and the intervention will therefore be particularly cost-effective in these patients.

Patients with both fatigue and depression are more disabled and generally have a worse prognosis over time^33^. Remitted patients with fatigue at baseline are assumed to have an increased risk of developing a new onset of depression^34^.

Besides fatigue, also the prognosis of depression and comorbid pain is poor compared with the prognosis for individuals with depression without pain ^35^. Pain with comorbid depression appears to be additive in terms of an increased number of medical visits and higher health care costs. In this study, both fatigue and pain are assessed to evaluate their possible relation with the overall outcome in terms of cumulative incidence of depression and total healthcare costs.

Both number of previous episodes and type of previous care are used for stratification allowing us to study these factors as potential effect modifiers.

An earlier first onset of depression (i.e. at a younger age) is associated with a poorer prognosis regarding the course of the depression (more recurrences, chronicity). One suggestion therefore is that early onset disorders typify a subset of patients with a distinct etiological profile in which genetic vulnerability and early developmental aspects play a larger role^36^. A large, recent Dutch study (NESDA) already confirmed that early onset disorders – apart from severity and duration – seem to result in poorer prognosis^37^.

The higher prevalence of depression in the lower socioeconomic strata (SES) suggests that there is a negative association between depression and socioeconomic status. The process linking SES and depression might be based on 2 personal resources: income and education. Higher-SES individuals are better endowed with such resources, leading to better health outcomes. A better health outcome (fewer recurrences) due to a higher level of education might be explained by higher levels of the patients’ self-efficacy for self-management of depression in these patients. Self-efficacy refers to the perceived ability to produce a desired action and captures both self-esteem (evaluation of own worth) and self-mastery (evaluation of self-command. Above average levels of self-efficacy is protective in the onset of depressive disorders^38^.

The hypotheses in this trial are that SES (in terms of attained education level) and depression are negatively correlated and that higher SES, via self-efficacy, is positively associated with better treatment response.

# 3. STUDY DESIGN

This study is a randomised controlled trial with randomisation at patient level. There will be two parallel groups to evaluate the costs and effects of nurse-led self-help + usual care for 134 primary care patients with remitted MDD versus 134 controls (usual care) at 3, 6, 9 and 12 months follow up. Stratification variables will be the number of previous episodes (cut-off point at 4 or more previous episodes in the last 5 years) and the type of treatment as usual received for the last episode (psychological intervention / AD / no care).

***Figure 1: Flowchart.***

**4. STUDY POPULATION**

## 4.1 Population (base)

The research assistant will select records in the participating primary care practices of subjects with long term AD use (ATC code) or depression (ICPC code P76) in the previous 5 years. From the list of eligible patients the PCP indicates patients that can be approached for a short information letter with contact information and first informed consent for global screening and receiving extensive information. A patient can reply by email, mail or telephone. For global screening, a patient is asked:

1) if he/she is known with depressive episodes, confirmed by a doctor

2) if these depressive episodes come with manic episodes (bipolar disorder, exclusion criterion) or not (unipolar disorder, inclusion-criterion)

3) if he/she experienced 2 or more depressive episodes in the last 5 years

4) if he/she is currently remitted

5) if he/she is willing to participate in a trial aimed at preventing the recurrence of depression

If a patient is potentially eligible for the trial based on the global screening, he/she will be sent an extensive information letter, informed consent and a response form. If a patient agrees to participate and signs informed consent the patient is checked by the research-assistant for definite in- and exclusion criteria. The aim is to execute the project in the primary care practices that already have mental health nurses working in their practices.

According to the Landelijk Informatie Netwerk Huisartsenzorg (LINH), the prevalence of ‘depression’ (ICPC; P76) is around 20.3 per 1000 registered patients (men plus women) aged 15-65 in primary care. An average primary care practice mostly consists of 2.500 patients, resulting in 50 depressed patients in total. Of these, according to literature, 85% experience a recurrence in the next 5 years. ^39^ The assumption therefore is that (50*0.85) 43 patients per practice will theoretically be eligible for screening.

##

## 4.2 Inclusion criteria

1. age 18-65 year
2. at least 2 confirmed previous MDD episodes with the SCID ^40^ in the last 5 years
3. current remission meaning:

a. a current score of <10 in the 17 item HRSD, in line with other prevention studies, ^14;15;41^

b. the last episode was at least 2 months ago (according to criteria DSM-IV)

c. the last episode was no longer than 2 years ago

1. fluent in Dutch

## 4.3 Exclusion criteria

1. current mania or hypomania or history of bipolar illness, according to record PCP
2. any current organic brain damage or psychotic disorder, according to record PCP
3. severe sensory disabilities

## 4.4 Sample size calculation

The trial is powered to detect a difference in the primary end term (cumulative recurrence rate) of 20%. To detect a 20% risk reduction of recurrence between the conditions in a 2 sided test at alpha (=0.05) and a power of 1-beta (=0.80), 107 patients in each condition are necessary. Compensating for loss to follow-up of 10% over the year (107+107/ 0.8=) 238 participants will be recruited.

# 5. TREATMENT OF SUBJECTS

**5.1 Investigational product/treatment**

## The investigational treatment in this trial is ‘nurse-led self-help’ based on PCT; this preventive cognitive therapy has been demonstrated to be protective in recurrent depression for a period of at least 2 to 5,5 years ^42^.

## Patients will be offered a detailed treatment manual of the therapy with literature, backgrounds and assignments. This self-help book will enable patients to follow the course of the therapy in their own homes, in their own time. Because experience has shown that patients may come across some difficulties along the course of this therapy, and remitted patients may lack motivation to complete the intervention there will be contact with the nurse on a regular base in order to encourage patients to work through the intervention. The nurses lend (minimal) support and do not engage in a therapeutic relationship with the patients. By implementing the intervention in this way the nurse-led self-help PCT is hypothesized to be (cost-) effective in comparison with usual care, readily accessible at the PCP level and will assume a personalised approach to the patients and their needs.

## Prior to the start of the therapy, a face-to-face meeting with the nurse is planned at the primary care practice (at a maximum of 30 minutes). This meeting involves motivational interviewing, psycho-education on (the course and treatment of) recurrent depression, and an introduction to the nurse-led self-help therapy on the basis of the treatment manual. Apart from this face-to-face meeting there is weekly telephone contact (at a maximum of 15 minutes), initiated by the nurse. During these telephone meetings patients are asked several questions based on a rather strict protocol. These questions comprise: 1) did you fill out Q-IDS-SR questionnaire? 2) did you read and understand the literature belonging to that week? 3) did you make the accompanying **assignments?** and 4) what difficulties did you experience in your assignments? After these 3 questions, patients are shortly prepared for next week’s literature and **exercises**. The contact is of a supportive and facilitating nature and not of a psychotherapeutic nature.

If a nurse notices depressive symptoms during a regular phone-contact or a patient brings up feeling depressed, the nurse emphasizes specific parts of the therapy in order for the patient to cope with these symptoms. Only in the case of a patient expressing suicidal symptoms, the PCP should be notified. These procedures are made explicit in the informed consent papers.

After each contact the nurse summarizes the conversation in a journal on the basis of a checklist. The checklist includes 6 questions:

1) Did the patient fill out the Q-IDS-SR questionnaire of that week?

2) Did the patient read and understand the literature belonging to that week: (yes/no)

- lack of time

- feeling too depressed

- too difficult/did not understand - didn’t feel like

- other …

3) Did the patient make the accompanying assignments: (yes/no)

- lack of time

- feeling too depressed

- too difficult/ did not understand - didn’t feel like

- other …

4) What difficulties did the patient experience? (open)

5) How long did the conversation take (in minutes)

6) What consecutive number is this telephone contact (1 / 2 / 3 / 4 / 5 / 6 / 7 / 8)

This journal is a way to both monitor and promote treatment integrity both on the side of the patient (did he/she read and apply the literature) and the nurse (did he/she go through all the questions). In addition, the contacts with the patients are randomly audiotaped and evaluated during supervision. Any adherence and/or competence issues will be checked this way.

## Nurses will deliver the nurse-led self- help. The face-to-face contacts take place in the primary care practice. The telephone conversations are conducted from the office. The nurses are trained by a professional from Dr. Bockting’s group. All practice nurses are experienced in offering cognitive therapy to depressed patients. It takes approximately 1 or 1,5 day to train the nurses. In total, around 15 nurses from 25 different practices will be trained.

## Treatment as usual involves usual care (i.e. standard/routine treatment, including no treatment), as typically provided by the PCP according to the Dutch PCP clinical guidelines (NHG-guidelines). There is no restriction on the use of pharmacotherapy, including the use of anti-depressants during the period form entry to end of follow-up.

Because patients either receive routine care (in the control condition) or an intervention that has been proven to be effective, there is no financial compensation, neither for patients, nor for nurses/PCP’s.

##

## 5.2 Use of co-intervention (if applicable)

*N/A*

## 5.3 Escape medication (if applicable)

*N/A*

# *6.* INVESTIGATIONAL MEDICINAL PRODUCT

*N/A*

# *7.* METHODS

## 7.1 Study parameters/endpoints

### 7.1.1. Main study parameter/endpoint

The primary outcome measure is the cumulative rate of recurrences meeting DSM-IV criteria for a major depressive episode (American Psychiatric Association, 1994) on the SCID ^40^ at 3, 6, 9, 12 and 15 months in both arms (expressed as relative risk ratios).

Quality of life is measured using the EuroQol ^43^ and 12-item Short Form Health Survey (SF-12)^44^. Costs are measured from a societal perspective using the TIC-P^45^. Costs of the intervention, other health care utilization, patients' out-of-pocket costs and costs due to productivity losses will be included in the economic evaluation. The EuroQol, SF-12 and TiC-P are assessed at 3, 6, 9, 12 and 15 months.

### 7.1.2. Secondary study parameters/endpoints

Secondary outcome is symptom severity as measured weekly during the course of the intervention with the ‘Quick-Inventory of Depressive Symptoms–Self Report’ questionnaire (Q-IDS-SR). At baseline the socio-economic and demographic characteristics are reported (age, gender, education, etc). Also a life-chart (recording a patient’s course of depressive episodes and treatments over the last 5 years) is completed at baseline.

Secondary outcomes will be measured at baseline and at 3,6,9,12 and 15 months follow-up: Q-IDS-SR, Four Dimension Symptom Questionnaire (4-DSQ, comorbid psychopathology), Fatigue Severity Scale (FSS, fatigue), Mac Gill Pain Questionnaire (MPQ, pain), General Self Efficacy Scale (GSES, self-efficacy), Medication Adherence Questionnaire (MAQ, medication adherence) and Nemesis Somatic Illnesses List. At 15 months a Client Satisfaction Questionnaire (CSQ-8) will be assessed in 20 ‘experimental’ patients who responded best, and 20 ‘experimental’ patients who responded worse (in terms of recurrences).

## 7.2 Randomisation, blinding and treatment allocation

Patients, who are eligible for the trial and who give informed consent for participation in the trial, will be randomized to a ‘nurse-led self-help plus usual care’ or ‘usual care alone’ therapy. Randomisation will take place at the level of the patient. Stratification variables will be the number of previous depressive episodes (cut off point at 4 or more episodes in the last 5 years) and the type of treatment received for the last recurrent episodes. It is not possible to blind patients or healthcare providers to the intervention in this study due to the nature of the intervention.

## 7.3 Study procedures

The research assistant checks in- and exclusion criteria. ‘Being remitted’ in this trial is defined as a score of < 10 on the 17-item Hamilton Depression Rating Scale. In order to assess the severity of residual symptoms a baseline, Q-IDS-SR score is calculated. In general, a score of 0-5 represents ‘normal/ no depression’ and a score 6-10 represents ‘mild depression’. A score of 11-15 indicates ‘moderate depression’, 16-20 ‘severe depression’ and 21-27 ‘very severe depression’.

After signing informed consent demographic data and the medical history are collected as part of routine care and in order to enable stratification. After randomisation, the nurse invites intervention subjects into the practice for an initial motivational face-to-face meeting about the course of depression and strategies to prevent recurrence. Also the nurse-led self-help therapy is explained.

Primary outcome is the (repeat) incidence of depressive disorder according to an independent and blinded Structured Clinical Interview for DSM-IV (SCID) ^40^ at 3, 6 ,9, 12 and 15 months. Secondary outcome is the Four Dimension Symptom Questionnaire (4-DSQ) ^31^, used every 3 months to assess co-morbid levels of self-reported distress, anxiety symptoms and levels of physical symptoms (somatisation). Satisfaction is measured with the Client/Patient Satisfaction Questionnaire (CSQ-8)^46^. The CSQ-8 is only assessed at 15 months in 20 ‘experimental’ patients who responded best, and 20 ‘experimental’ patients who responded worse (in terms of recurrences). Satisfaction is best linked to effectiveness this way.

Generic health-related quality of life is measured using both the EuroQol^43^ and SF-12^44^. QALYs will be calculated based on the EuroQol using the Dutch tariff by Lamers et al^47^ . Cost data related to health care uptake will be measured using the TIC-P ^45^. Medical costs that will be assessed include costs related to the intervention, medication use, hospital admissions, and contacts with healthcare professionals. Unit resource use will be multiplied by their appropriate integral cost prices^47^. Cost data stemming from absenteeism and presenteeism from paid work will be collected with the PRODISQ. Costs of productivity losses will be estimated using the friction cost method. In a secondary analysis, the human capital will also be used to estimate productivity losses. All questionnaires are assessed at 3, 6, 9, 12 and 15 months.

SES is measured by asking for level of education. Also items on self-efficacy in preventing depression which predict future risk for depressive disorder are obtained. These items are assessed at baseline, 6 and 12 months.

For an overview of assessments at baseline, during the intervention and during follow-up, see table 1. Collecting of data through questionnaires and analyzing the data is done by the main researcher and the research assistant who are not blinded.

Casefinding in this trial should not be a problem as these high risk patients are often seen by the PCP and are rather easily diagnosed with recurrent depression in the long term. Hence, inclusion should not be endangered.

**7.3.1 Informed consent**

Eligible patients that are found through a search in the database receive a global study information letter with 4 questions, including if the patient agrees upon receiving detailed information about the study. If the patient is interested in participating then the participant will contact the researcher or the researcher will contact the patient after 2 weeks and checks interest in participation. Subsequently, the researcher checks that the participant signs an informed consent for screening.

When global screening is positive, patients are sent a complete study information letter and an informed consent for participation in the trial. Definite inclusion / and exclusion criteria are checked through telephone-interviews and when the informed consent for participation in the trial is received the patient will enter the trial.

Consenting patients are asked to provide information about their socio-economic and demographic background and are assessed for their eligibility in more detail using semi-structured clinical interviews (SCID-I, HRSD-17 by phone) and self-report questionnaires.

## 7.4 Withdrawal of individual subjects

Participants can withdraw from treatment or from the study at any time. Nevertheless we ask those who withdraw from any arm if they are willing to attend all the remaining research appointments or at least to provide minimal data.

### 7.4.1 Specific criteria for withdrawal

Subjects who relapse into depression during the course of the intervention will be encouraged to continue the therapy. Besides, possible adjustments in the actual treatment will be started after consultation of the PCP. Patients who don’t want to continue, will be referred by the nurse to specific parts of the self-help in order to promote ongoing prevention of relapse. Subjects in the non-experimental group who relapse will continue their care as usual.

## 7.5 Replacement of individual subjects after withdrawal

Patients who discontinue the intervention are not replaced (and analysis is conducted in agreement with the intention to treat principle). Patients who skipped a measurement at one time point are approached for the next (thus causing only wave non-response), unless they withdraw.

##

## 7.6 Follow-up of subjects withdrawn from treatment

We will encourage those who withdraw from any arm if they are willing to attend all the remaining research appointments or at least to provide minimal data and are encouraged to continue participation. Besides, early drop-outs will be contacted by phone in order to ask for their motivation to quit (exit-conversation). Data of withdrawn patients will remain included in the statistical analysis.

## 7.7 Premature termination of the study

Premature termination of the study is possible in case of advice to do so from the monitoring committee. If the study is prematurely terminated the Medisch Etische Toetsings Commissie (METC), Bevoegde Instantie (BI) and College ter Beoordeling Geneesmiddelen (CBG) will be notified.

**Table 1 Overview of questionnaires**

| Measure | Description | T0 | T1 | T2 | T3 | T4 | T5 |
| --- | --- | --- | --- | --- | --- | --- | --- |
| ***Interviews*** |  | | | | | | |
| SCID-I | DSM-IV-TR Axis I  Disorders | + | + | + | + | + | + |
| HRSD-17 | Depressive symptoms  and severity | + | + | + | + | + | + |
| ***Self report measures*** |  | | | | | | |
| Q-IDS-SR | Depressive symptoms | + | + | + | + | + | + |
| EQ-5D | Quality of life | + | + | + | + | + | + |
| SF-12 | Quality of life | + | + | + | + | + | + |
| TIC-P | Direct / indirect costs | + | + | + | + | + | + |
| 4DSQ | Comorbid psychopathology | + | + | + | + | + | + |
| FSS | Severity of fatigue | + | + | + | + | + | + |
| MPQ-DLV | Severity / evaluation of pain | + | + | + | + | + | + |
| General self-efficacy scale | Self-efficacy | + | + | + | + | + | + |
| MAQ | Medication Adherence | + | + | + | + | + | + |
| Nemesis Somatic illnesses list | List of somatic disorders | + |  | + |  |  | + |
| CSQ-8 | Satisfaction |  |  |  |  |  | + |
| Life-chart | Course of symptoms | + |  |  |  |  |  |

T0=baseline, T1=3 month, T2=6 months, T3 = 9 months, T4= 12 months, T5 = 15 months

# 8. SAFETY REPORTING

## 8.1 Section 10 WMO event

In accordance to section 10, subsection 1, of the WMO, the investigator will inform the subjects and the reviewing accredited METC if anything occurs, on the basis of which it appears that the disadvantages of participation may be significantly greater than was foreseen in the research proposal. The study will be suspended pending further review by the accredited METC, except insofar as suspension would jeopardise the subjects’ health. The investigator will take care that all subjects are kept informed.

## 8.2 Adverse and serious adverse events

Adverse events are defined as any undesirable experience occurring to a subject during the study, whether or not considered related to the experimental treatment. All adverse events reported spontaneously by the subject or observed by the investiga­tor or his staff will be recorded.

A serious adverse event is any untoward medical occurrence or effect that at any dose:

- results in death;
- is life threatening (at the time of the event);
- requires hospitalisation or prolongation of existing inpatients’ hospitalisation;
- results in persistent or significant disability or incapacity;
- is a congenital anomaly or birth defect;
- is a new event of the trial likely to affect the safety of the subjects, such as an unexpected outcome of an adverse reaction, lack of efficacy of an IMP used for the treatment of a life threatening disease, major safety finding from a newly completed animal study, etc.

In this trial, serious adverse events related to the investigational treatment are relapse into depression with or without suicidal thoughts or the start of any other psychopathology (i.e. anxiety disorder). Hospitalisation might be necessary depending on the severity of the symptoms. No specific somatic disorders are expected during the course of this intervention.

All SAEs will be reported through the web portal *ToetsingOnline* to the accredited METC that approved the protocol, within 15 days after the sponsor has first knowledge of the serious adverse reactions. SAEs that result in death or are life threatening should be reported expedited.

The expedited reporting will occur not later than 7 days after the responsible investigator has first knowledge of the adverse reaction. This is for a preliminary report with another 8 days for completion of the report.

### 8.2.1. Suspected unexpected serious adverse reactions (SUSAR)

*N/A*

### 8.2.2. Annual safety report

*N/A*

## 8.3 Follow-up of adverse events

All adverse events will be followed until they have abated, or until a stable situation has been reached. Depending on the event, follow up may require additional tests or medical procedures as indicated, and/or referral to the PCP or a medical specialist.

## Data Safety Monitoring Board (DSMB)

N/A

# *9.* STATISTICAL ANALYSES

## 9.1 Descriptive statistics

Standard descriptive methods (e.g., frequencies and percentages or mean ± SD) will be used to summarize the demographic and clinical features of the controls and the patients who received the intervention.

All calculated *P* values are 2 sided; statistical significance will be set at *P* <.05.

**9.2 Analysis of primary clinical outcome**

The primary outcome measure is the cumulative recurrence over the respective follow-up times meeting DSM-IV criteria for a major depressive episode (American Psychiatric Association, 1994) on the SCID-I at 3, 6, 9, 12 and 15 months in both arms (yielding relative risk ratios). When adding the intervention is superior then the recurrence rate in this condition should be smaller than in the control group. Therefore cumulative recurrence rate ratios under a Poisson regression model are obtained. The occurrence of recurrence (current or since the last assessment point) will be assessed by trained research assistant who is not blind to treatment condition.

Number-needed-to-be-treated (NNT) will be calculated as the inverse of the risk difference (RD).

All analyses will be conducted in agreement with the intention-to-treat principle, meaning all patients attending at least the face-to-face meeting at the start of the therapy will be included as obtained under the Expectation-Maximization algorithm (EM) algorithm^48^ at 3, 6, 9, 12 and 15 months follow-up. To gauge the robustness of the outcomes, this analysis will be repeated while using a multiple-imputation approach.

The above analyses will take into account that patients are ‘nested’ within primary care practices. To that end, we will conduct design-based data-analyses with Stata’s (Version 9.2 for Windows) survey commands.

**9.3 Economic evaluation**

For the valuation of health care utilization standard prices published in the Dutch costing guidelines will be used^49^. Medication use will be valued using prices of the Royal Dutch Society for Pharmacy. Unit prices of the different intervention elements will be calculated using a bottom-up approach. Costs for personnel, patient materials, rental of practice spaces and other expenses will be included in these calculations. Costs and effects are not discounted.

Missing cost and effect data will be imputed using multiple imputation according to the MICE algorithm developed by van Buuren^50^. Costs typically have a highly skewed distribution. Policy makers want to have information on the difference in mean total costs between the two treatment groups to be able to estimate the total health care budget needed for a specific condition. ^51^ Therefore, bias-corrected and accelerated bootstrapping with 5000 replications will be used to calculate 95% confidence intervals around the mean difference in total costs between the treatment groups.

The economic evaluation will be conducted from a societal perspective, both as a cost-effectiveness analysis (CEA, with depression-free person years as the clinical end term) and as a cost-utility analysis (CUA, with incremental costs per quality adjusted life years (QALY) gained) as the clinical end-term. Bootstrapping will be used to estimate the uncertainty surrounding the ICERs which will be graphically presented on cost-effectiveness planes. Cost-effectiveness acceptability curves and net monetary benefits will also be calculated. Cost-effectiveness acceptability curves show the probability that collaborative care is cost-effective in comparison with usual care for a range of different ceiling ratios thereby showing decision uncertainty.^52^

These analyses help to answer the question whether the intervention offers good value for money.

**9.4 Analysis of secondary clinical outcomes**

Moderator analyses will be conducted for some demographic variables. Subgroups that show particularly good response to the intervention will be identified by regressing HRDS depression severity on the interaction term of treatment and clinical characteristics of the patients as measured at baseline. Examples of other characteristics are number of previous depressive episodes, treatment preference, age of onset of first depression, self-efficacy in preventing depression, pain and fatigue, social economic status other demographic characteristics like gender, marital and occupational status and age.

**9.5 Analyses of secondary economic outcomes**

Analogous to the moderator analyses for clinical end-terms, an incremental net benefit regression (INBR) analysis will be conducted to addresses the research question in what groups the intervention is likely to be particularly cost-effective. The same set of predictor variables (see above) will also be used in these INBR analyses.

The Incremental Net Benefit will be calculated as Eλ – C. The first term is the number of units of effectiveness gained multiplied by an unknown quantity (λ) what one is willing to pay (WTP) for a unit of effectiveness gained. Because λ is unknown, we will use a likely WTP-range. The product term is subtracted by the costs yielding the net benefit expressed in monetary terms. Incremental net-benefits will be obtained using a regression analysis approach and these help to identify moderators (sub-groups) for which the intervention is particularly cost-effective^53^.

## 9.6 Interim analysis (not applicable)

An interim analysis can be performed during a running clinical trial for reason of safety and efficacy. The main reason for interim analysis on efficacy is to verify whether important clinical differences exist between the treatment group and the control group have become evident. The main reason for interim analysis on safety are performed to guarantee the safety of the patients during the trial. Because efficacy is best to be expected only after 12 months of follow-up and safety-issues are hardly to be expected in this non-medicine trial, an interim analysis will not be performed. Any possible issues with blinding and privacy are prevented this way.

#

# 10. ETHICAL CONSIDERATIONS

## 10.1 Regulation statement

This study will be conducted according to the principles of the Declaration of Helsinki

(Version 2004) and with the Medical Research Involving Human Subjects Act (WMO). By

signing this protocol the investigators commit themselves to conduct this study in according

to Good Clinical Practice (GCP).

## 10.2 Recruitment and consent

Only when a patient fulfils both inclusion- and exclusion criteria, the subject will be included.

## 10.3 Objection by minors or incapacitated subjects (if applicable)

*N/A*

## 10.4 Benefits and risks assessment, group relatedness

Risks in this trial are very low because usual care is permitted in both arms. Regarding benefits; PCT has proven effective in preventing recurrences in patients with MDD. Besides, the authors of this protocol have good experiences with offering PCT in patients with MDD. This study should be performed in this specific group of patients with a history of recurrences because the effect of the prevention therapy is highest in this high-risk group.

## 10.5 Compensation for injury

The sponsor/investigator has a liability insurance which is in accordance with article 7, subsection 6 of the WMO.

The sponsor (also) has an insurance which is in accordance with the legal requirements in the Netherlands (Article 7 WMO and the Measure regarding Compulsory Insurance for Clinical Research in Humans of 23rd June 2003). This insurance provides cover for damage to research subjects through injury or death caused by the study.

1. € 450.000,-- (i.e. four hundred and fifty thousand Euro) for death or injury for each subject who participates in the Research;
2. € 3.500.000,-- (i.e. three million five hundred thousand Euro) for death or injury for all subjects who participate in the Research;
3. € 5.000.000,-- (i.e. five million Euro) for the total damage incurred by the organisation for all damage disclosed by scientific research for the Sponsor as ‘verrichter’ in the meaning of said Act in each year of insurance coverage.

The insurance applies to the damage that becomes apparent during the study or within 4 years after the end of the study.

## 10.6 Incentives (if applicable)

N/A

# 11. ADMINISTRATIVE ASPECTS AND PUBLICATION

## 11.1 Handling and storage of data and documents

Data are collected in digital patient record forms. The construction and maintenance of this

electronic Case Report Form (eCRF) is handled by the department of data management of

the VU Medical Centre/ EMGO. The department of data management will build and manage a database based on these eCRF’s. Study data are handled confidentially and anonymously

and in accordance with the Dutch personal data protection act (de wet bescherming

persoonsgegevens). Subjects have unique identification codes (study number). Study

related data that are not directly linked to clinical patient management are stored using that

study number.

## 11.2 Amendments

Amendments are changes made to the research after a favourable opinion by the accredited METC has been given. All amendments will be notified to the METC that gave a favourable opinion. Non-substantial amendments will not be notified to the accredited METC and the competent authority, but will be recorded and filed by the sponsor.

## 11.3 Annual progress report

The sponsor/investigator will submit a summary of the progress of the trial to the accredited METC once a year. Information will be provided on the date of inclusion of the first subject, numbers of subjects included and numbers of subjects that have completed the trial, serious adverse events/ serious adverse reactions, other problems, and amendments.

## 11.4 End of study report

The investigator will notify the accredited METC of the end of the study within a period of 8 weeks. The end of the study is defined as the last patient’s last visit.

In case the study is ended prematurely, the investigator will notify the accredited METC, including the reasons for the premature termination.

 Within one year after the end of the study, the investigator/sponsor will submit a final study report with the results of the study, including any publications/abstracts of the study, to the accredited METC.

## 11.5 Public disclosure and publication policy

# Results of the study will be made public in peer-reviewed scientific journals.

# 12. REFERENCES

(1) Murray CJ, Lopez AD. Regional patterns of disability-free life expectancy and disability-adjusted life expectancy: global Burden of Disease Study. *Lancet* 1997; 349(9062):1347-1352.

(2) Ormel J, Petukhova M, Chatterji S, Aguilar-Gaxiola S, Alonso J, Angermeyer MC et al. Disability and treatment of specific mental and physical disorders across the world. *Br J Psychiatry* 2008; 192(5):368-375.

(3) Vos T, Haby MM, Barendregt JJ, Kruijshaar M, Corry J, Andrews G. The burden of major depression avoidable by longer-term treatment strategies. *Arch Gen Psychiatry* 2004; 61(11):1097-1103.

(4) Paykel ES. Continuation and maintenance therapy in depression. *Br Med Bull* 2001; 57:145-159.

(5) Van Loenen AC r. Farmacotherapeutisch kompas. College voor Zorgverzekeringen 2003. 2003. Ref Type: Journal (Full)

(6) Viguera AC, Baldessarini RJ, Friedberg J. Discontinuing antidepressant treatment in major depression. *Harv Rev Psychiatry* 1998; 5(6):293-306.

(7) Bockting CL, ten Doesschate MC, Spijker J, Spinhoven P, Koeter MW, Schene AH. Continuation and maintenance use of antidepressants in recurrent depression. *Psychother Psychosom* 2008; 77(1):17-26.

(8) Meijer WE, Heerdink ER, Leufkens HG, Herings RM, Egberts AC, Nolen WA. Incidence and determinants of long-term use of antidepressants. *Eur J Clin Pharmacol* 2004; 60(1):57-61.

(9) ten Doesschate MC, Bockting CL, Koeter MW, Schene AH. Predictors of nonadherence to continuation and maintenance antidepressant medication in patients with remitted recurrent depression. *J Clin Psychiatry* 2009; 70(1):63-69.

(10) Kaymaz N, van OJ, Loonen AJ, Nolen WA. Evidence that patients with single versus recurrent depressive episodes are differentially sensitive to treatment discontinuation: a meta-analysis of placebo-controlled randomized trials. *J Clin Psychiatry* 2008; 69(9):1423-1436.

(11) Piek E, van der Meer K, Nolen WA. Guideline recommendations for long-term treatment of depression with antidepressants in primary care--a critical review. *Eur J Gen Pract* 2010; 16(2):106-112.

(12) van Schaik DJ, Klijn AF, van Hout HP, van Marwijk HW, Beekman AT, de HM et al. Patients' preferences in the treatment of depressive disorder in primary care. *Gen Hosp Psychiatry* 2004; 26(3):184-189.

(13) Hollon SD, Ponniah K. A review of empirically supported psychological therapies for mood disorders in adults. *Depress Anxiety* 2010; 27(10):891-932.

(14) Vittengl JR, Clark LA, Dunn TW, Jarrett RB. Reducing relapse and recurrence in unipolar depression: a comparative meta-analysis of cognitive-behavioral therapy's effects. *J Consult Clin Psychol* 2007; 75(3):475-488.

(15) Bockting CL, Schene AH, Spinhoven P, Koeter MW, Wouters LF, Huyser J et al. Preventing relapse/recurrence in recurrent depression with cognitive therapy: a randomized controlled trial. *J Consult Clin Psychol* 2005; 73(4):647-657.

(16) Bijl RV, Ravelli A. Psychiatrische morbididteit, zorggebruik en zorgbehoefte. Resultaten van de Netherlands Mental Health Survey and Incidence Study. Tijdschrift voor Gezondheidswetenschappen 76, 446-57. 1998. Ref Type: Journal (Full)

(17) Le LA, Despiegel N, Francois C, Duru G. Can discrete event simulation be of use in modelling major depression? *Cost Eff Resour Alloc* 2006; 4:19.

(18) Teasdale JD, Segal ZV, Williams JM, Ridgeway VA, Soulsby JM, Lau MA. Prevention of relapse/recurrence in major depression by mindfulness-based cognitive therapy. *J Consult Clin Psychol* 2000; 68(4):615-623.

(19) Ma SH, Teasdale JD. Mindfulness-based cognitive therapy for depression: replication and exploration of differential relapse prevention effects. *J Consult Clin Psychol* 2004; 72(1):31-40.

(20) Stant AD, TenVergert EM, Kluiter H, Conradi HJ, Smit A, Ormel J. Cost-effectiveness of a psychoeducational relapse prevention program for depression in primary care. *J Ment Health Policy Econ* 2009; 12(4):195-204.

(21) den Boer PC, Wiersma D, Van den Bosch RJ. Why is self-help neglected in the treatment of emotional disorders? A meta-analysis. *Psychol Med* 2004; 34(6):959-971.

(22) Gregory RJ, Canning SS, Lee TW, Wise JC. Cognitive bibliotherapy for depression: A meta-analysis. *Professional Psychology-Research and Practice* 2004; 35(3):275-280.

(23) McKendree-Smith NL, Floyd M, Scogin FR. Self-administered treatments for depression: a review. *J Clin Psychol* 2003; 59(3):275-288.

(24) Van't Hof E, Cuijpers P, Stein DJ. Self-help and Internet-guided interventions in depression and anxiety disorders: a systematic review of meta-analyses. *CNS Spectr* 2009; 14(2 Suppl 3):34-40.

(25) Marrs RW. A meta-analysis of bibliotherapy studies. *Am J Community Psychol* 1995; 23(6):843-870.

(26) Newman MG, Erickson T, Przeworski A, Dzus E. Self-help and minimal-contact therapies for anxiety disorders: Is human contact necessary for therapeutic efficacy? *J Clin Psychol* 2003; 59(3):251-274.

(27) Spek V, Cuijpers P, Nyklicek I, Riper H, Keyzer J, Pop V. Internet-based cognitive behaviour therapy for symptoms of depression and anxiety: a meta-analysis. *Psychol Med* 2007; 37(3):319-328.

(28) Hunkeler EM, Meresman JF, Hargreaves WA, Fireman B, Berman WH, Kirsch AJ et al. Efficacy of nurse telehealth care and peer support in augmenting treatment of depression in primary care. *Arch Fam Med* 2000; 9(8):700-708.

(29) Mynors-Wallis LM, Gath DH, Day A, Baker F. Randomised controlled trial of problem solving treatment, antidepressant medication, and combined treatment for major depression in primary care. *BMJ* 2000; 320(7226):26-30.

(30) Bosmans JE, de Bruijne MC, de Boer MR, van Hout H, van Steenwijk P, van Tulder MW. Health care costs of depression in primary care patients in The Netherlands. *Family Practice* 2010; 27(5):542-548.

(31) Terluin B, van Marwijk HW, Ader HJ, de Vet HC, Penninx BW, Hermens ML et al. The Four-Dimensional Symptom Questionnaire (4DSQ): a validation study of a multidimensional self-report questionnaire to assess distress, depression, anxiety and somatization. *BMC Psychiatry* 2006; 6:34.

(32) Paykel ES. Partial remission, residual symptoms, and relapse in depression. *Dialogues Clin Neurosci* 2008; 10(4):431-437.

(33) Joyce J, Hotopf M, Wessely S. The prognosis of chronic fatigue and chronic fatigue syndrome: a systematic review. *QJM* 1997; 90(3):223-233.

(34) Skapinakis P, Lewis G, Mavreas V. Temporal relations between unexplained fatigue and depression: longitudinal data from an international study in primary care. *Psychosom Med* 2004; 66(3):330-335.

(35) Geerlings SW, Twisk JW, Beekman AT, Deeg DJ, van TW. Longitudinal relationship between pain and depression in older adults: sex, age and physical disability. *Soc Psychiatry Psychiatr Epidemiol* 2002; 37(1):23-30.

(36) Kendler KS, Fiske A, Gardner CO, Gatz M. Delineation of two genetic pathways to major depression. *Biol Psychiatry* 2009; 65(9):808-811.

(37) Penninx BW, Nolen WA, Lamers F, Zitman FG, Smit JH, Spinhoven P et al. Two-year course of depressive and anxiety disorders: Results from the Netherlands Study of Depression and Anxiety (NESDA). *J Affect Disord* 2011.

(38) Van Voorhees BW, Paunesku D, Kuwabara SA, Basu A, Gollan J, Hankin BL et al. Protective and vulnerability factors predicting new-onset depressive episode in a representative of U.S. adolescents. *J Adolesc Health* 2008; 42(6):605-616.

(39) Mueller TI, Leon AC, Keller MB, Solomon DA, Endicott J, Coryell W et al. Recurrence after recovery from major depressive disorder during 15 years of observational follow-up. *Am J Psychiatry* 1999; 156(7):1000-1006.

(40) First MB, Gibbon M, Spitzer RL, Williams JBW. Structured Clinical Interview for DSM-IV Axis I Disorders, Clinician Version (SCID-CV). Washington, D.C: American Psychiatric Press, Inc; 1996.

(41) Bockting CL, Elgersma HJ, van Rijsbergen GD, de JP, Ormel J, Buskens E et al. Disrupting the rhythm of depression: design and protocol of a randomized controlled trial on preventing relapse using brief cognitive therapy with or without antidepressants. *BMC Psychiatry* 2011; 11:8.

(42) Bockting CL, Spinhoven P, Wouters LF, Koeter MW, Schene AH. Long-term effects of preventive cognitive therapy in recurrent depression: a 5.5-year follow-up study. *J Clin Psychiatry* 2009; 70(12):1621-1628.

(43) EuroQol group. EuroQol--a new facility for the measurement of health-related quality of life. The EuroQol Group. *Health Policy* 1990; 16(3):199-208.

(44) Ware J, Jr., Kosinski M, Keller SD. A 12-Item Short-Form Health Survey: construction of scales and preliminary tests of reliability and validity. *Med Care* 1996; 34(3):220-233.

(45) Hakkaart-van Roijen L, Van Straten A, Donker M, et al. Manual:Trimbos/iMTA Questionnaire for Costs Associated with Psychiatric Illness (in Dutch). Rotterdam: Erasmus University; 2002.

(46) Larsen DL, Attkisson CC, Hargreaves WA, Nguyen TD. Assessment of client/patient satisfaction: development of a general scale. *Eval Program Plann* 1979; 2(3):197-207.

(47) Lamers LM, Stalmeier PF, McDonnell J, Krabbe PF, van Busschbach JJ. [Measuring the quality of life in economic evaluations: the Dutch EQ-5D tariff]. *Ned Tijdschr Geneeskd* 2005; 149(28):1574-1578.

(48) Dempster AP, Laird NM, Rubin DB. Maximum Likelihood from Incomplete Data via the EM Algorithm. *Journal of the Royal Statistical Society Series B (Methodological)* 1977; 39(1):1-38.

(49) Hakkaart-van Roijen L, Tan SS, Bouwmans CAM. Handleiding voor kostenonderzoek. Methoden en standaard kostprijzen voor economische evaluaties in de gezondheidszorg ed. Rotterdam: Instituut voor Medical Technology Assessment iov CZV; 2010.

(50) van Buuren S, Oudshoorn CGM. Multivariate imputation by chained equations. MICE V1.0 user's manual. Leiden: TNO Preventie en Gezondheid; 2000.

(51) Thompson SG, Barber JA. How should cost data in pragmatic randomised trials be analysed? *BMJ* 2000; 320(7243):1197-200.

(52) Fenwick E, O'Brien BJ, Briggs A. Cost-effectiveness acceptability curves--facts, fallacies and frequently asked questions. *Health Econ* 2004; 13(5):405-415.

(53) Hoch JS, Rockx MA, Krahn AD. Using the net benefit regression framework to construct cost-effectiveness acceptability curves: an example using data from a trial of external loop recorders versus Holter monitoring for ambulatory monitoring of "community acquired" syncope. *BMC Health Serv Res* 2006; 6:68.
